# Older adults’ experiences of health seeking in rural areas in low- and middle-income countries: a systematic review of qualitative studies

**DOI:** 10.1093/heapol/czaf061

**Published:** 2025-10-28

**Authors:** Ziyue Wang, Xiaochen Ma, Can Su, Yihang Zhang, Xiang Zou, Mobolanle Balogun, Howard Bergman, Xiaoyun Liu, Nadia Sourial, Isabelle Vedel

**Affiliations:** Department of Family Medicine, Faculty of Medicine and Health Sciences, McGill University, 5858 Côte-Des-Neiges Road, 3rd Floor, Montreal, Quebec H3S 1Z1, Canada; China Centre for Health Development Studies, Peking University, No 38. Xueyuan Rd, Haidian District, Beijing 100191, China; School of Public Health, Peking University, No 38. Xueyuan Rd, Haidian District, Beijing 100191, China; School of Public Health, Peking University, No 38. Xueyuan Rd, Haidian District, Beijing 100191, China; Department of Medical Humanities, Southeast University, Liberal Arts Building, No. 2 Dongnandaxue Road, Jiangning District, Nanjing 211189, China; Department of Community Health and Primary Care, College of Medicine, University of Lagos, Idi Araba, PMB 12003, Lagos, Nigeria; Department of Family Medicine, Faculty of Medicine and Health Sciences, McGill University, 5858 Côte-Des-Neiges Road, 3rd Floor, Montreal, Quebec H3S 1Z1, Canada; China Centre for Health Development Studies, Peking University, No 38. Xueyuan Rd, Haidian District, Beijing 100191, China; Department of Health Management, Evaluation and Policy, École de Santé Publique (School of Public Health), Université de Montréal, 7101 Avenue du Parc, 3rd floor, Montreal, Quebec, H3N 1X9, Canada; Department of Family Medicine, Faculty of Medicine and Health Sciences, McGill University, 5858 Côte-Des-Neiges Road, 3rd Floor, Montreal, Quebec H3S 1Z1, Canada

**Keywords:** rural and remote health, ageing, health-seeking behaviours, low- and middle-income countries (LMICs), systematic review, thematic synthesis

## Abstract

The global aged population is expected to reach 2.1 billion by 2050 and ∼40% of them will live in rural areas of low- and middle-income countries (LMICs). This systematic review aims to synthesize the qualitative literature on rural older adults’ experiences of health-seeking in LMICs as well as explore the factors that influence their experiences during their health-seeking journeys. We searched Embase, MEDLINE, PsycINFO, and CINAHL to identify studies published from 1 January 2002 to 31 December 2024 (PROSPERO registration ID: CRD42022358813). We used a thematic synthesis approach to analyse included studies. Among the 19 studies with 28 articles and 484 participants included, 16 were rated as high quality, 9 as moderate quality, and 3 as weak quality. We identified four primary analytic domains associated with their experiences in health-seeking journeys: (i) individual—depicting the inner world of rural older adults; (ii) interpersonal—navigating the rural social network; (iii) organizational—navigating the rural health care systems, and; (iv) community and macrosystems—economy, society, and public policy in rural areas. Rural older adults in LMICs have experienced unique and multi-level challenges in seeking care. To overcome these challenges, rural older adults demonstrated resilience and creativity (e.g. utilizing informal institutions), to navigate their health-seeking journey. Future research should aim to better understand the resilience and agency in local older adults’ health-seeking experiences and provide constructive solutions to overcome identified barriers to care.

Key messages

- The review identified multi-level healthcare barriers for rural older adults in LMICs, including economic hardship, inadequate infrastructure, and perceived worthlessness, which result from long-term neglect by formal economic, social, and health institutions.
- To address these challenges, the studies included in this review highlight older adults’ reliance on social networks, alternative care, and community-based resources to mitigate gaps in formal healthcare and access to alternative care solutions.
- Collaboration with alternative providers may offer a viable approach to improving healthcare access, reducing disparities, and creating culturally appropriate and sustainable health solutions in rural LMICs.
- Future studies should examine how older adults actively shape healthcare experiences and offer practical solutions that align with local cultural and economic contexts, rather than presenting ‘deficit-oriented’ perspectives on the health systems in rural LMICs.

## Introduction

Population ageing represents a significant demographic shift with important implications for health systems worldwide, particularly in low- and middle-income countries (LMICs) (Beard et al. 2016, Fried 2016, World Health Organization 2023).^1^ However, substantial rural–urban disparities in socioeconomic development and health system capacity remain, and many rural areas retain characteristics more similar to lower-middle-income settings. While it can be viewed as an indicator of developmental success, including improved healthcare access and economic growth, this population transition presents unique challenges for rural health systems (United Nations Department of Economic and Social Affairs Population Division 2020). Currently, ∼30% of older adults globally reside in rural areas of LMICs, with projections suggesting this will rise to 40% by 2050 (Alhassan Issahaku and Neysmith 2013, Fried 2016, Miranda et al. 2019, Skinner et al. 2020). These regions often face compounded challenges, including limited healthcare infrastructure, workforce shortages, and geographic barriers that disproportionately affect older rural populations.

In addition to the growing ageing population in rural areas of LMICs, the health-seeking behavior (HSB) of older adults in these areas is becoming a particularly prominent issue. Several structural and sociocultural factors have hindered older adults’ access to adequate health care services in rural areas of LMICs. First, most LMICs are not prepared for their ageing populations, especially in rural areas. The absence of pensions and benefits places a disproportionate burden on vulnerable and underserved populations in rural areas (Agyeman et al. 2019, Camarero and Oliva 2019, Agyemang-Duah et al. 2019a, Agyemang-Duah et al. 2020, Hamiduzzaman et al. 2021a, Wu et al. 2022). Second, as the concept of HSB—‘a process of how patients engage with the health system within certain social systems, cultural norms and system constraints’ (Mackian et al. 2004)—suggests, older patients’ health-seeking in rural areas is shaped by many nuanced socioecological factors that may not be directly linked to healthcare settings (Elnegaard et al. 2015, Zhang et al. 2020). For example, unlike the health system in high-income countries (HICs) or in metropolitan areas of LMICs, which are more dominated by formal, Western healthcare institutions, the rural health care system in LMICs is more pluralized, including multiple combinations of formal (e.g. hospitals, clinics) and informal (e.g. alternative care providers) institutions (Brundisini et al. 2013, Skinner et al. 2020, Parsons et al. 2021, Kielmann et al. 2022, Ohta et al. 2022, Kalita et al. 2023). In this way, health-seeking strategies of rural older adults are also more diversified than those of their urban or HIC counterparts, but very few articles on health-seeking have focused on these rural-specific issues and their policy implications. Finally, contrary to the traditional assumptions of health care providers and researchers, rural people are not always passive; instead, they use their own strategies to shape their daily lives and their navigation of the health system (Ku 2003, Tsai 2007).

To better understand the nuances of older adults’ HSBs in rural areas of LMICs, a systematic review of qualitative studies is well-suited to capture the depth and context of individuals’ lived experiences and perceptions. Unlike quantitative approaches that primarily describe prevalence or associations, qualitative synthesis allows us to uncover how older adults interpret their health needs, navigate structural barriers, and make decisions within specific sociocultural and health system contexts (Creswell and Poth 2016). Although there have been several previous systematic reviews of qualitative rural studies in HICs (Brundisini et al. 2013, Parsons et al. 2021, Golembiewski et al. 2022, Kielmann et al. 2022, Ohta et al. 2022), to the best of our knowledge, there is no qualitative synthesis for older adults in rural LMICs. To address this gap, this study aims to (i) synthesize the qualitative literature on rural older adults’ experiences of health-seeking in LMICs and (ii) examine the underlying factors shaping these experiences through the lens of established theoretical frameworks.

## Methods

This review follows the Enhancing Transparency in Reporting the Synthesis of Qualitative Research (ENTREQ) statement guidelines (Tong et al. 2012) and the Preferred Reporting Items for Systematic Reviews and Meta-analyses (PRISMA) statement (Moher et al. 2009) (supplementary Appendices S7 and S8, see online supplementary material). All materials were coded and analyzed using NVivo (version 12, QSR International).

### Search strategy and selection criteria

#### Search strategy

We searched Embase, MEDLINE, PsycINFO, and CINAHL. PubMed/MEDLINE indexes >5200 biomedical journals from >150 countries, and incorporates >3000 journals published in LMICs through the MEDLINE selection process. Embase covers >8500 journals, including 2900 not indexed in PubMed, with particular strength in European and Asian literature. Embase’s emphasis on pharmacological and device-related research also provides valuable insights into informal healthcare utilization patterns. PsycINFO is an authoritative database for behavioral and psychological sciences, which indexes >2500 psychology journals, covering 99% of peer-reviewed journals in the discipline since 1887. This database is critical for capturing the psychosocial dimensions of HSB. CINAHL is the leading database for nursing, which indexes >1300 journals, and was used to identify qualitative studies published between 1 January 2002, and 31 December 2024. As this study was originally designed in 2022, the 20-year timeframe (2002–2022 initially, now extended to 2024) provides sufficient historical perspective while capturing recent developments in LMIC healthcare systems. In addition, the early 2000s marked increased scholarly attention to ageing populations in LMICs, making this an appropriate starting point for capturing relevant qualitative studies. The detailed search strategy of each database is listed in supplementary Appendix 1 in the online supplementary material.

#### Inclusion and exclusion criteria

Areas being studied
Eligible studies were required to explicitly address people’s experiences (e.g. the process of making or receiving an appointment, cleanliness of facilities, waiting times, the information provided, and interactions with healthcare professionals), views, or feelings (e.g. patient’s perception, interpretations of treatment, and satisfaction with care) when navigating their health care system. These experiences could occur within or outside of the health care setting (e.g. the influence of illness/injury on their family, friends, and other social activities). Empirical studies focusing exclusively on supply-side perspectives (e.g. health systems, care providers), or studies assessing only perceived quality or satisfaction with health care, were not eligible.
Participants
The populations of the included studies featured populations with at least 80% participants aged ≥60 years (a commonly used threshold for defining older age in LMICs) living in rural areas of LMICs [see the World Bank's list (World Bank 2025)]. The participants of included studies could include older adults, their caregivers, or both. To ensure the generalizability of the study, we imposed no disease-specific restrictions on health-seeking journeys.
Type of studies
Eligible studies employed qualitative or mixed methods. Non-empirical studies (e.g. review, editorial, guidelines) were excluded. We considered only English-language peer-reviewed articles and did not incorporate grey literature.

#### Screening and data extraction

According to a preset hierarchical screening protocol (supplementary Appendix 2, see online supplementary material), two reviewers (CS & YZ) independently reviewed all records by titles and abstracts. If reviewers had difficulty determining the eligibility of a study, they would assess the full article based on the same eligibility criteria. Differences among the two reviewers in screening, data extraction, coding, and quality assessment were resolved by consensus or referred to the third and fourth reviewers (ZW & XM). Publications using the same dataset were considered as a single study. Inter-reviewer reliability was estimated using kappa scores. Two researchers (CS & YZ) also independently collected characteristics of interest following the preset data extraction protocol (supplementary Appendix 3, see online supplementary material). Supplementary Appendix 4 (see online supplementary material) provides a detailed list of the relevant characteristics of included studies. Screening was performed using Endnote 9 and data extraction was carried out with Excel 2019. Following the practice of Thomas and Harden (2008) in thematic synthesis, we copied all the text labeled as ‘results’ or ‘findings’ from included manuscripts for coding and synthesis.

#### Assessment of study quality

We used the Mixed Methods Appraisal Tool (MMAT version 2018) for quality assessment (Hong et al. 2018), which was developed and validated for the critical appraisal of studies with diverse designs. A score of 1 or 0 was assigned to each criterion based on its presence or absence. The MMAT quality score for each article was calculated by dividing the total number of points obtained by the total possible points. Each article was categorized as having weak (≤0.50), moderate-weak (0.51–0.65), moderate-strong (0.66–0.79), or strong (≥0.80) study quality based on its MMAT quality score (supplementary Appendix 5, see online supplementary material). We did not exclude studies based on their quality level as we wanted to have a comprehensive understanding of patients’ experiences (Noyes et al. 2024).

### Thematic synthesis

We followed the three-step process of thematic synthesis (Thomas and Harden 2008, Dennison et al. 2020). First, two independent researchers (CS & YZ) conducted free line-by-line coding of the findings of primary studies. Then the two reviewers independently organized these ‘free codes’ to construct descriptive themes. In the third stage, the research team further analyzed and reorganized themes into new analytical themes with a conceptual framework inspired by two models, the institutional adaptation model (Azari and Smith 2012, Helmke and Levitsky 2012) and the social-ecological model of health (SEM) (Bronfenbrenner 2000, Golden et al. 2015) (see a detailed description of the frameworks below).

Our coding framework was guided by Helmke & Levitsky's institutional adaptation model (Azari and Smith 2012, Helmke and Levitsky 2012) and the SEM (Bronfenbrenner 2000, Golden et al. 2015). The details of these two frameworks are shown in supplementary Appendix 6 (see online supplementary material). Given the important role of informal institutions in rural areas of LMICs, we used the institutional adaptation model, which was originally developed by political scientists Helmke and Levitsky, to explain how formal and informal institutions shape and constrain individual and collective action in developing countries. Helmke and Levitsky define informal institutions as socially shared, usually unwritten rules enforced outside officially sanctioned channels, while formal institutions are rules and procedures created and enforced through recognized authorities such as courts, legislatures, bureaucracies, constitutions, laws, and organizational rules (Fukuyama 1996, Helmke and Levitsky 2012, Bian 2019). In the institutional adaptation model, the interaction between formal and informal institutions depends on effectiveness and goals: they are “complementary” when motivations align and formal institutions are effective; “accommodating” when motivations differ but formal institutions are effective; “substitutive” when informal institutions pursue similar goals where formal ones are weak; and “competing” when objectives diverge and formal institutions lack strength. Building on this institutional lens, we also incorporated the SEM, which is widely applied in public health research and offers a complementary perspective by emphasizing the complex interplay among individual, interpersonal, organizational, community, and societal factors that shape health and social behaviors. Unlike approaches that focus mainly on individual characteristics or proximal influences, SEM enables a broader analysis of contextual drivers of behavior and has been widely used in health services research among underserved populations (Ma et al. 2017, Kahn et al. 2022).

To inform our interpretation using these two models, we followed the five-stage process for applying frameworks in qualitative synthesis described in the Cochrane–Campbell Handbook for Qualitative Evidence Synthesis (Noyes et al. 2024). We also used a sensitivity analysis to assess the potential impact of the study quality on our evidence synthesis. We removed all codes from manuscripts with weak study quality (MMAT score ≤ 0.50) to see the influence of weak-quality studies on our final results.

#### Ethical approval and registration

Since this study was based on publicly available data, ethical approval was not required. The review protocol was registered at PROSPERO (CRD42022358813).

#### Declaration of use of generative AI and AI-assisted technologies in the writing process

During the preparation of this article, the authors used GPT-3.5 (OpenAI, San Francisco, CA, USA) in order to improve the readability of this article. After using this tool/service, the authors reviewed and edited the content as needed and take full responsibility for the content of the publication.

## Results

### Studies’ characteristics (see Fig. 1)

The search yielded 2997 records. After excluding ineligible studies based on the title, abstract, or full-text articles, a total of 19 original studies involving 28 publications and 484 unique participants were included in the data extraction and synthesis (kappa = 0.56) (Rotchford et al. 2002, Liu et al. 2006, Hossen and Westhues 2010, Hossen and Westhues 2011, Lewallen et al. 2011, Zhang et al. 2011, Hossen and Westhues 2012, Li et al. 2012, Van Hoi et al. 2012, Hossen et al. 2013, Long and Li 2016, Nielsen et al. 2017, Schatz et al. 2018, Agyeman et al. 2019, Naidoo and Van Wyk 2019, Zou and Nie 2019, Agyemang-Duah et al. 2019a, Agyemang-Duah et al. 2019b, Agyemang-Duah et al. 2020, Hamiduzzaman 2020, Maciel et al. 2020, Zou et al. 2020, Wang and Qi 2021, Hamiduzzaman et al. 2021b, Hamiduzzaman et al. 2021b, Hamiduzzaman et al. 2021c, Hamiduzzaman et al. 2022, Wu et al. 2022). Table 1 and supplementary Appendix 2 present the characteristics of these 19 studies. All studies (100%) included older adults, while less than one-third of them (six studies, 31.6%) also included caregivers. The largest share of studies (6/19, 31.6%) was from China, followed by Bangladesh (4/19, 21.1%), Ghana (2/19, 10.5%), South Africa (2/19, 10.5%), and Uganda (2/19, 10.5%). Most of the included studies covered one or more chronic diseases (15/19, 78.9%). The spectrum of disease was quite broad, including eye diseases (Rotchford et al. 2002, Lewallen et al. 2011, Zhang et al. 2011), cardiovascular diseases (Zou and Nie 2019, Zou et al. 2020, Hamiduzzaman et al. 2021b), diabetes (Li et al. 2012, Nielsen et al. 2017), stroke (Li et al. 2012), bronchitis (Hamiduzzaman et al. 2021c), cancer (Zou et al. 2020), dementia (Agyeman et al. 2019, Zou et al. 2020), and HIV/AIDS (Schatz et al. 2018). In terms of research settings, 11 of the 19 studies collected data in community settings, while the other eight studies recruited their participants in clinical settings. Most studies used a narrative research approach (Creswell and Poth 2016) (11/19, 57.9%), and very few researchers used other qualitative study approaches like ethnography. Eleven studies cited pre-existing theories, models, or frameworks to frame their studies such as interpretivism, critical theories (e.g. feminist theories, Habermas’ theory of communicative action), the socio-ecological model, and the social determinants of health framework.

**Figure 1.**
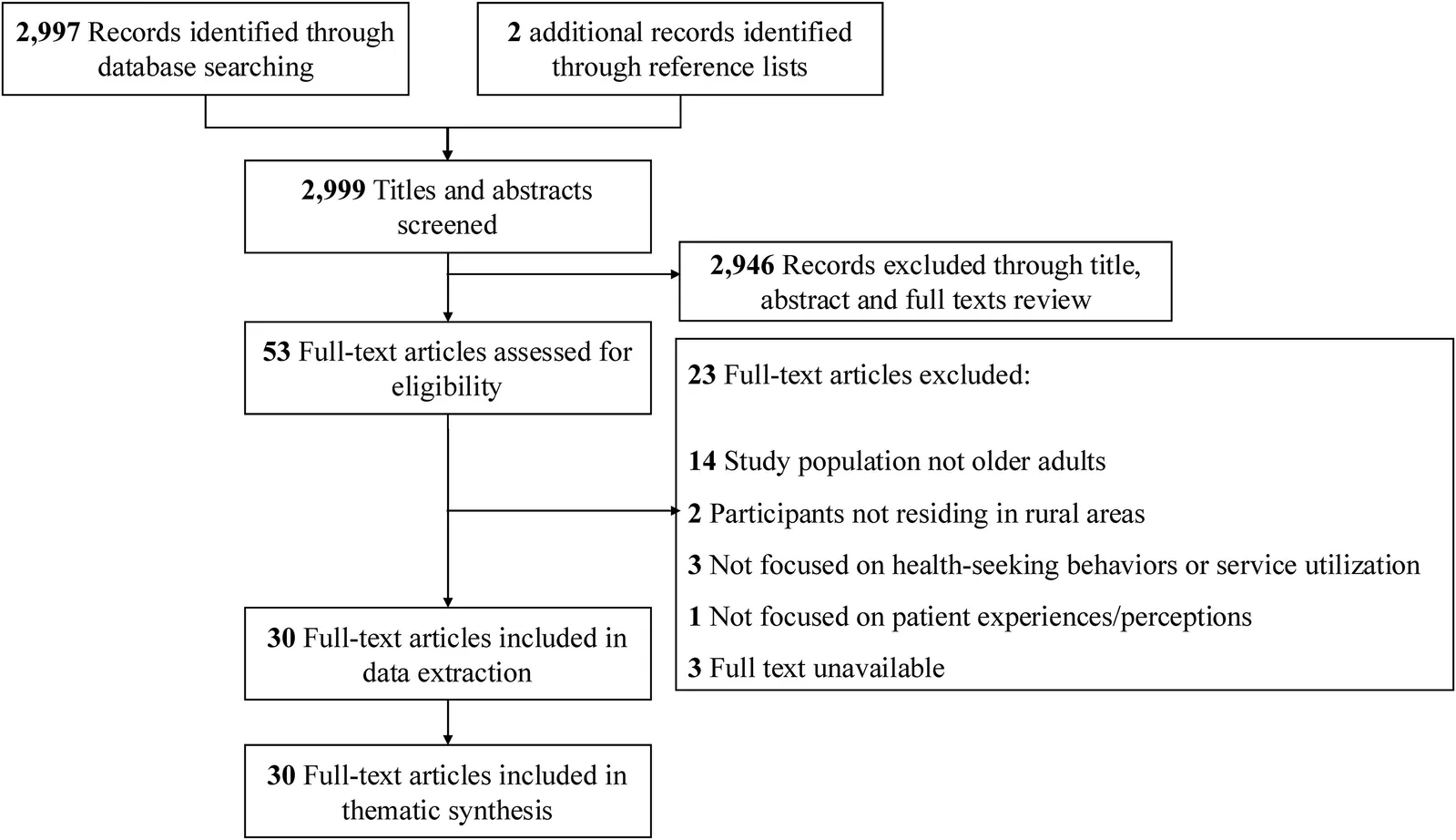
Flow chart.

**Table 1.** Summary of study characteristics (*n* = 19).

No.	Author, year conducted^a^	Location	Participant type (patient, or caregiver)	Diseases/health conditions studied	Number of participants	Age distribution, years	Sex and/or gender distribution	Research setting	Theoretical framework	Study design
1	Agyeman-Duah, 2015	Kintampo, Ghana	Patients and caregivers	Dementia	28 in total (10 patients and some of their co-residents)	Mean 84.9; range 73–100	Patients: 6 females (60%), caregivers: 15 females (100%)	Clinical setting	NA	Case study
2	Agyemang-Duah, 2018	Atwima Nwabiagya District, Ghana	Patients and caregivers	NA	Patients: 30, caregivers: 15	Patients: 65–69: 9; 70–74: 6; 75–79: 3; 80–84: 4; 85–89: 3; ≥90: 5	Patients: 23 females (77%), caregivers: 15 females (100%)	Clinical setting	Interpretivist paradigm	Convergent mixed method study design (qualitative component: narrative research)
3	Hamiduzzaman, 2015	Sylhet district, Bangladesh	Patients	88% (22/25) experienced at least two diseases and 68% (17/25) reported at least three, over the year of data collection	25	Mean 72; Range 60–100	25 female (100%)	Clinical setting	A blended critical social model based on Habermas’ Theory of Communicative Action and Honneth's Theory of Recognition and Misrecognition	Narrative research
4	Hamiduzzaman, 2015	Sylhet district, Bangladeshi	Patients	Self-reported seasonal symptoms	25	Mean 72 (SD: 10.2)	65 Female (100%)	Clinical setting	NA	Mixed-methods study
5	Hossen, 2006	Sherpur district, Bangladesh	Patients	NA	17	∼60–75	17 Female (100%)	Community setting	a social-determinants-of-health perspective	Phenomenology (a feminist phenomenological approach)
6	Le, 2007	Bavi, Vietnam	Four FGDs: One FGD—six older people. One FGD—six representatives of households with older people. Other two FGDs—caregivers.	NA	6	>60	3 Females (50%)	Community setting	NA	Convergent mixed method study design
7	Lewallen, 2007	Kilimanjaro Region, Tanzania	Patients	Bilaterally blind (Visual acuity< 3/60) from posterior segment causes, including glaucoma, other optic nerve disease, and various retinal conditions	19	Mean: 77 (SD = 15)	8 Females (42%)	Community setting	NA	Narrative research
8	Liu *et al*., 2006	Shaanxi, China	Patients	NA	20	Mean 65.72; range 60–72	Females (55%)	Community setting	Participatory action research model and critical social theory	Participatory action research
9	Maciel, 2018	Rio Grande do Sul, Brazil	Patients	NA	19	≥60	NA	Clinical setting	NA	Descriptive and exploratory qualitative study
10	Naidoo, 2018	KwaZulu-Natal, South Africa	Patients	NA	28	>60	19 Females (67.9%)	Clinical setting	NA	Interpretative exploratory design
11	Nielsen, 2013	Kasese District, Uganda	Patients and caregivers	Type 2 diabetes	10	45–60: 2 ≥ 60: 8	4 Females (40%)	Clinical setting	Therapy management group	Case study
12	Rotchford *et al*. 2002	South Africa	patients	Cataract	20	Mean 78; range 56–88	13 Females (65%)	Community setting	NA	Narrative research
13	Schatz, 2015	Kalungu District, Uganda	patients	2 Groups with HIV; 2 groups with NCD; 5 groups without specific diagnosis	9 Groups (7–8 people in each group)	>60	Men: 5 groups; Women: 4 groups	Community setting	Three-Delay Model	Narrative research
14	Long and Li, 2016	Shandong, China	Patients and caregivers	NA	Patients: 12; caregivers: 12	Patients: mean 72; range 56–82 Caregivers: median (range): 53 (29–79)	Patients: 7 female (58.3%); caregivers: 8 females (66.7%)	Community setting	Bourdieu's dialectical model	Narrative
15	Li *et al*., 2012	Shandong, China	Patients and caregivers	Heart disease: 3 Diabetes: 2 Stroke: 2 Bronchitis: 1 Hypertension: 1 Other:4	Patients: 12; caregivers: 12	Patients: mean 72, range 56–82. Caregivers: median (range): 53 (29–79)	Patients: 7 female (58.3%); caregivers: 8 females (66.7%)	Community setting	NA	Narrative
16	Wang, 2019	Shandong, China	Patients	Living with disability	13	≥60	7 Female (53.8%)	Community setting	Theory of welfare pluralism	Narrative
17	Wu, 2020	Shandong, China	Patients	Healthy without illness 3; with minor illness 2; with mild chronic illness 4; with serious chronic illness 7	16	60–70: 8; 70–80: 6; 80–100: 2	8 Females (50%)	Community setting	NA	Narrative research
18	Zhang *et al*. 2011	Guangdong, China	Patients	Cataract (best-corrected visual acuity of <6/18 in either eye)	20	Mean 72.7 ± 6.1 years	14 Females (70%)	Community setting	NA	Narrative research
19	Zou, 2016	Guangdong, China	Patients and caregivers	Multiple chronic health problems: 13/20 had cardiovascular disease and dementia, 4 suffered from advanced cancer or other terminal condition	20 Patients and their caregivers	>60	NA	Clinical setting	Feminist ethical framework	Ethnography

^a^If the year the study was conducted is unavailable, the publication year is listed.

Of the 28 papers included, three were of ‘weak’ quality (10.7%), 9 were of ‘moderate-strong’ quality (32.1%) and the remaining 16 papers were of ‘strong’ quality (57.2%) (supplementary Appendix 5). By removing all codes from studies with weak quality, we did not see any change in our final analytical framework, which means low-quality studies have fewer unique themes and contribute less to the thematic synthesis.

### Result of thematic synthesis

**Figure 2.**
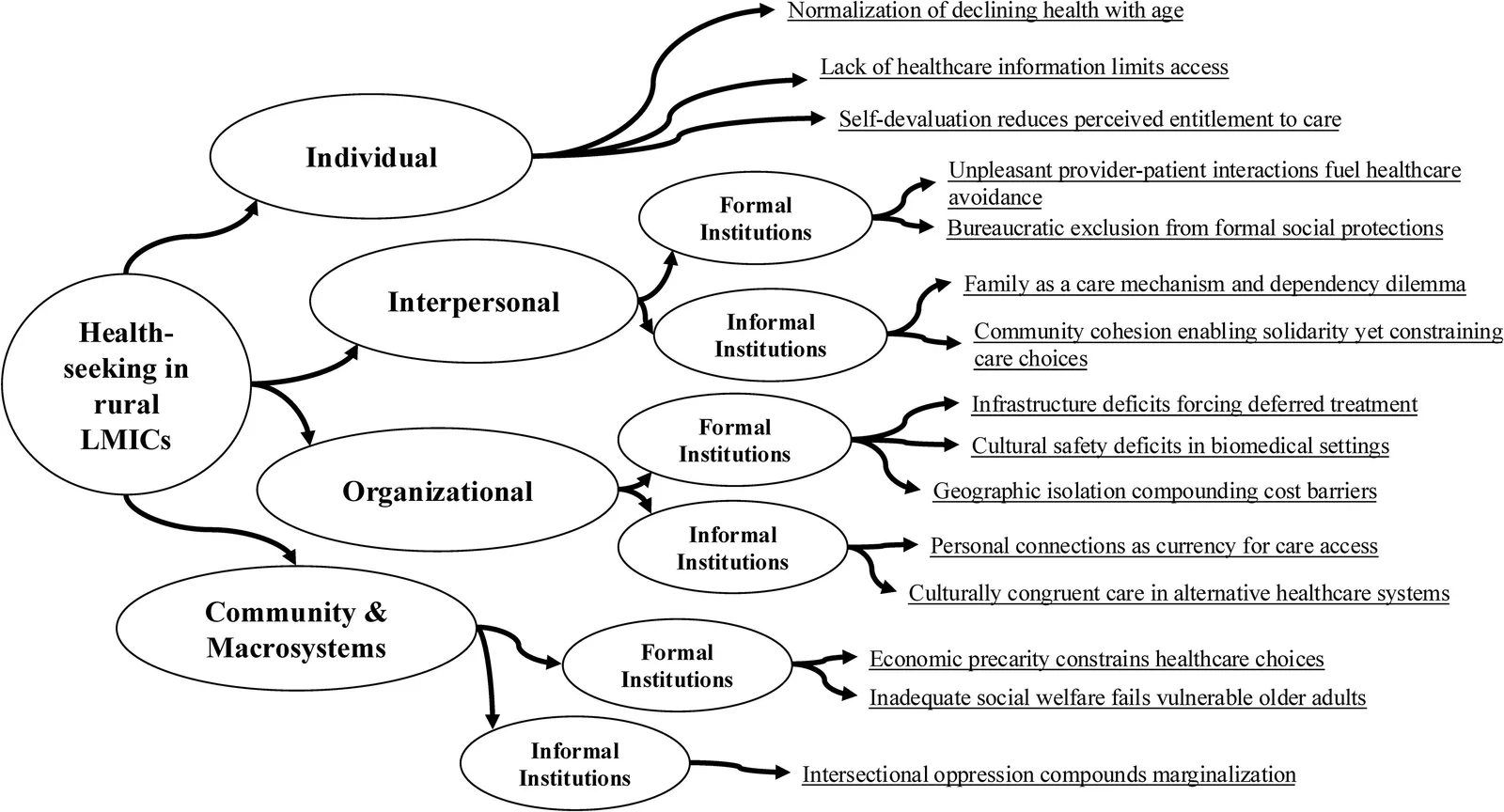
Summary of themes.

Our thematic synthesis identified four domains associated with rural older adults’ experiences of health-seeking in LMICs (Table 2 and Fig. 2). These domains and underlying themes are described in detail below and typical quotes from the participants are given.

**Table 2.** Key themes identified in this analysis with example quotes.

Domain	Description	Analytic theme	Example quote
1. Individual: depicting the inner world of rural Older patients	The influence of rural environment on elderly patients’ conceptions of illness and health in their mind.	Normalization of declining health with age	‘When you get old you are bound to see less, and other things like joint pain (bathjor) or weakness (durbolota) are gifts of old age.’ (Hossen et al. 2013)
		Lack of healthcare information limits access	‘As I cannot read the prescription, I have to rely on the pharmacists who sell medications… I must pay as they ask for.’ (Hamiduzzaman et al. 2021b)
		Self-devaluation reduces perceived entitlement to care	‘The village committee gave me some money, like 60–70 yuan a year. But I can still work. We cannot ask the state for stuff. That’s wrong.’ (Long and Li 2016)
2. Interpersonal: navigating the rural social network	The characteristics of the social network in rural areas that affect the experience of or ability to access health care services.	Informal relationship: family as a care mechanism and dependency dilemma.	‘After seeing the doctor, it is already evening when you return home. Who is going to do your housework?’ (Hossen and Westhues 2011)
		Informal relationship: community cohesion enabling solidarity yet constraining care choices.	‘Those who have no sons or daughters are taken care of by others, and arrangements are made to go to a nursing home. Otherwise, people will laugh at you.’ (Wang and Qi 2021)
		Formal relationship: unpleasant provider–patient interactions fuel healthcare avoidance.	‘They (health workers) saying ‘You are just finishing/wasting our medicine! Your period [of survival] is over!’.’ (Schatz et al. 2018)
		Formal relationship: bureaucratic exclusion from formal social protections.	‘I went to the local chairman’s house for several times to put my name in the list of old age allowance. I requested them, but they did not include my name in the list.’ (Hamiduzzaman et al. 2021a)
3. Organizational: navigating the rural health care systems	Patient experiences of navigating rural health care systems and organizations to access timely, appropriate care.	Formal institutions: infrastructure deficits forcing deferred treatment.	‘I went to the hospital every day and they were asking me to come next day at each time, as the test equipment was not available.’ (Hamiduzzaman 2020)
		Formal institutions: cultural safety deficits in biomedical settings.	‘Women do not want to share their personal problems or gynaecological problems with male doctors… They like to share everything with female doctors.’ (Hamiduzzaman et al. 2021a)
		Formal institutions: geographic isolation compounding cost barriers.	‘There are no public healthcare facilities in our village. I have to go for a long way for accessing healthcare centers… There are no public transports in this road so that we have to use privately owned vehicles. It costs more.’ (Hamiduzzaman et al. 2022)
		Informal institutions: personal connections as currency for care access.	‘Tom was apparently aware of the advantages of having relations inside the hospital as he had sold part of his land to finance his son’s education as a nurse.’ (Nielsen et al. 2017)
		Informal Institutions: culturally congruent care in alternative healthcare systems.	‘We go to a mohila (female) kabiraj. She talks with us, prepares medicines, explains everything properly and nicely. She never charges us too much.’ (Hossen and Westhues 2012)
4. Community and macrosystems: economic, society, and public policy in rural areas	Patients’ perceptions of health-seeking journey in the context of the broader socioeconomic and cultural environment in rural areas.	Formal institutions: economic precarity constrains healthcare choices.	‘When they prescribe drugs, we are unable to get money to buy, this is making it difficult for us in terms of healthcare utilization’ (Agyemang-Duah et al. 2019b).
		Formal institutions: inadequate social welfare fails vulnerable older adults.	‘The insurance covers some of the drugs. There are some drugs that are not cover by the insurance and with this the aged have to do some top up.’ (Agyemang-Duah et al. 2019a)
		Informal institutions: intersectional oppression compounds marginalization.	‘My husband had a lot of assets, but I do not have anything now. I have no savings or property, so I am valueless for people.’ (Hamiduzzaman et al. 2021c)

## Individual: depicting the personal world of rural older adults

### Normalization of declining health with age

Participants in 14 studies described their understanding of health and diseases. Rural people in LMICs usually understand health as the absence of symptoms or mild symptoms that do not interfere with their ability to work. They recognize diseases as a normal phenomenon of aging. With this belief, many rural older adults tended to underestimate the severity of their diseases. As one participant stated: ‘It is normal for an older person like me to have some pain, discomfort, or other chronic illness… I would say for my age I am healthy since I can do my stuff by myself without any assistance’ (Hossen et al. 2013). Participants with religious beliefs also associate their diseases with god’s will or other supernatural causes, and even develop a sense of fatalism (Hossen and Westhues 2012, Hamiduzzaman et al. 2021a). They usually tolerate the distress, pray, or use self-medication to relieve the symptoms, and are unlikely to accept aggressive treatments like surgery.

### Lack of healthcare information limits access

Participants in five studies mentioned that limited health-related information has impeded their HSB. They usually have difficulty obtaining information about the availability and cost of treatments, how to enroll in health insurance, and how to file a claim. For example, in a study in Uganda, some participants did not know of the availability of free diabetes treatment at the public hospital (Nielsen et al. 2017). In another study in South Africa, people living with cataracts were surprised at the low price of surgery in hospital (Rotchford et al. 2002).

### Self-devaluation reduces perceived entitlement to care

Seven studies talked about the self-devaluation of participants. In many rural communities, there is an unfavorable cultural narrative towards older adults’ care, including how rural dwellers understand themselves (e.g. self-devaluation), their sense of entitlement (e.g. viewing themselves ‘unworthy of care and treatment’) (Zou and Nie 2019), their relationship with the state (e.g. the discourse of self-reliance in terms of state/citizen responsibilities in care), and the subjective efficacy of treatment (e.g. downplaying the significance of treatment and postponing necessary treatment). As one participant from rural China said: ‘The village committee gave me some money, like 60–70 yuan a year. But I can still work. We cannot ask the state for stuff. That’s wrong’ (Long and Li 2016).

## Interpersonal: navigating the rural social network

### Formal relationships

#### Unpleasant provider–patient interactions fuel healthcare avoidance

Participants in 11 of the 19 studies cited unpleasant experiences when they talked with rural care providers. Ageism (Naidoo and Van Wyk 2019), gender and class discrimination (Nielsen et al. 2017, Hamiduzzaman et al. 2021c), being impatient (Agyemang-Duah et al. 2020), lack of respect (Hamiduzzaman et al. 2021b), verbal abuse (Agyemang-Duah et al. 2019b), and asking for a bribe (Lora-Wainwright 2011) were the most common complaints. Older adults and caregivers in nine studies also described their frustration with long waiting times for consultation. Living in a rural location had already added considerable travel time to an appointment, which fueled the frustration felt when patients had to wait in the office: ‘All of us have to wait, and they can tell us to come back next day… We are in our 70 s. Imagine …’ (Naidoo and Van Wyk 2019).

#### Bureaucratic exclusion from formal social protections

We found very few descriptions (two out of 19 studies) of formal interpersonal relationships other than provider–patient relationships (e.g. with government officials, Non-Governmental Organizations, and volunteers). Many older adults are reluctant to seek help from the government because they believe that their peasant identity makes them inferior, uncivilized, and unworthy of care (‘We are not workers or cadres. How would we deserve better?’) (Long and Li 2016) and many of them have never heard of NGOs or volunteer teams (Wang and Qi 2021). Individuals who seek assistance from these organizations also face unfavorable outcomes. As one Bangladeshi woman said: ‘I went to the local chairman’s house [locally elected representative] for several times to put my name in the list of old age allowance. I requested them, but they did not include my name in the list’ (Hamiduzzaman et al. 2021c).

### Informal relationships

#### Family as a care mechanism and dependency dilemma

The barriers and facilitators associated with family relations in rural environments were noted in various ways (14 studies). Older adults in rural areas of LMICs usually have a big family and they are highly dependent on their families for health care. As one participant from rural China described: ‘I live in the same village as my young brother’s son, who can drive and has received a good education. I would ask for him to accompany me to seek healthcare services when I am sick’ (Wu et al. 2022). However, being blood-related does not always guarantee unconditional support and care. In some cases, family members tend to prioritize attention and care for their children over older individuals. Older patients have to actively avoid becoming a burden on their families. An older woman in rural Bangladesh said: ‘You go to the hospital, buy a ticket, and wait for several hours to see the doctor…. After seeing the doctor, it is already late in the evening when you return home. Who is going to do your housework?’ (Hossen and Westhues 2011).

#### Community cohesion enabling solidarity yet constraining care choices

Having a close neighborhood is another prominent feature for rural residents compared to their urban counterparts. Close-knit communities can provide financial and emotional support, and they help older adults navigate the healthcare system. However, in certain cases, community norms and traditional values might limit the choice of care for older adults and their caregivers. For example, in some places, paying for care services is incompatible with local culture. Seeking formal healthcare would give their adult children a bad reputation in their community (Wang and Qi 2021). Moreover, people living with stigmatized health conditions (e.g. sexually transmitted infections, HIV/AIDS) were also under a high threat of social rejection and exclusion in rural communities (Hossen and Westhues 2010).

## Organizational: navigating the rural health care systems

### Formal institutions

#### Infrastructure deficits forcing deferred treatment

Older adults and caregivers in 14 studies complained of the lack of professionals, facilities, medication, and equipment in rural health facilities, particularly within the formal biomedical health system. One participant shared his experience: ‘I went to the hospital every day and they were asking me to come next day at each time, as the test equipment was currently not available’ (Hamiduzzaman 2020). In addition, rural health systems in LMICs are not well prepared for care of older adults and very few geriatricians practice in rural areas.

#### Cultural safety deficits in biomedical settings

In five studies, participants expressed a desire for greater cultural awareness and sensitivity among health care professionals working in the formal biomedical health system. Some participants cited general concerns about being a rural resident, including feeling belittled by clinicians, providers’ lack of gender/cultural awareness, dialect/language barriers, and miscommunication. For instance, gender is an extremely sensitive issue for religious women, as one Bangladeshi woman said: ‘I feel lojja [shame] to talk to a male doctor about mayali [female] problems…You can talk about mathabetha [headache] to a male doctor, but how can you show your book [breast] to a male doctor?’ (Hossen and Westhues 2010).

#### Geographic isolation compounding cost barriers

A common complaint cited in 11 studies was that living in rural areas compounds the already high cost of health care. Limited health resources in rural areas, difficult road conditions, and high travel fees are the main drivers for these additional costs. One participant noted: ‘There are no public healthcare facilities in our village… There is no public transport on this road so that we have to use privately owned vehicles. It costs more’ (Hamiduzzaman et al. 2022).

### Informal institutions

#### Personal connections as currency for care access

In the face of all the above-mentioned difficulties in accessing care within the formal health system, rural people have also developed their own strategy: fostering personal connections inside the local health care system to receive high-quality and affordable care. For example, an older person with diabetes had sold part of his land to ﬁnance his son’s education as a nurse so that he could develop a social connection in the local health system (Nielsen et al. 2017). In another study, researchers found that people in rural China only ‘consulted doctors from nearby villages when they had connections to them, or when these doctors were recommended by neighbors or relatives.’ They even tried to use the researchers as ‘insiders’ to find a trustworthy doctor for them (Lora-Wainwright 2011).

#### Culturally congruent care in alternative healthcare systems

Given the limited resources and unpleasant experiences of the formal biomedical health system care in rural areas of LMICs, alternative care plays a very important role in these areas. In seven studies, participants mentioned their choice between biomedical care and alternative care (e.g. spiritual medical and traditional healer). Participants agreed that alternative care in rural areas was cheaper, more accessible, provided more emotional comfort, and sometimes provided effective solutions to their health issues. Rural older individuals have a preference for alternative care due to three main factors. (i) Better quality of care and people-centered care. One participant described his experience of seeing a traditional healer: ‘The other day I went to the kabiraj [traditional healer]. I gave her two chickens… If I go to hospital, it takes time and money. I don’t have cash’ (Hossen and Westhues 2012). (ii) Belief in the effectiveness of alternative medicine. Alternative care providers share a similar cultural background and beliefs with patients: ‘We go to a mohila [female] kabiraj. She talks with us, prepares medicines, explains everything properly and nicely’ (Hossen and Westhues 2012). (iii) Better accessibility of care. Alternative care providers are more accommodating: ‘She (alternative care provider) never charges us too much. If it is midnight you can call her. She does not mind.’ (Hossen and Westhues 2012). Nevertheless, older adults realized that alternative care is not always reliable: ‘Nowadays, I still go to see the moulovi [faith healers] ﬁrst. If it does not work … wait 2 or 3 days … then go to the hospital’ (Hossen et al. 2013).

## Community and macrosystems: economy, society, and public policy in rural areas

### Formal institutions

#### Economic precarity constrains healthcare choices

Economic hardship is a common theme in rural communities. Older adults face significant disadvantages in the marketplace, and lack of money is one of the most important barriers to health care (mentioned in all 19 studies). Most of the rural older adults have no formal income and they depend on their children to pay medical bills. As one caregiver said: ‘The last time I took my mother to the hospital, I needed to borrow before I was able to send her. Due to lack of money, I always have to delay in seeking healthcare for my mother who is an older person’ (Agyemang-Duah et al. 2019a). In this situation, participants of nine studies mentioned competing financial priorities in their household (e.g. medical costs for the old, ceremonial expenses, childcare expenses, and other family members’ expenditures). Here is a typical quote from a family caregiver in Ghana: ‘As you can see these days money is difficult to get… all of my other siblings are married and have children to take care of … they pay school fees and other things…’ (Agyeman et al. 2019).

#### Inadequate social welfare fails vulnerable older adults

In 11 studies, people complained about meagre pensions, inadequate health insurance coverage, and limited social welfare. Participants described trouble obtaining payment for necessary specialty care because of the narrow scope of health insurance coverage in rural areas. As a health care provider from Ghana mentioned: ‘The insurance covers some of the drugs. There are some drugs that are not covered by the insurance’ (Agyemang-Duah et al. 2019b). Some countries have introduced social welfare programs to reduce the medical costs of older populations, such as the old age monthly allowance in Bangladesh (Hamiduzzaman et al. 2021a) and the Livelihood Empowerment Against Poverty program (LEAP) (Agyemang-Duah et al. 2019a) in Ghana. However, most of these social programs are either insufficient to cover high medical costs, have very restrictive eligibility criteria, or are not financially sustainable in the long-term (Wu et al. 2022).

### Informal institutions

#### Intersectional oppression compounds marginalization

Participants in seven studies reported multiple sociocultural disadvantages in rural areas, such as ageism, gender discrimination, education, social hierarchy, and religion, that hindered their health-seeking activities. These adversities are particularly relevant in a rural setting because farmers in most LMICs have little political power in an inequitable social system. A representative study from rural Bangladesh that focused on the experiences of older women explained how the intersectionality of multiple social adversities shaped their healthcare decisions. In rural Bangladesh, older women face enormous socioeconomic adversities including gender- and age-based social practices, economic deprivation, religious beliefs, and educational inequality that restrict the mobility of women. These factors reinforce the social roles of rural older women as incompetent, worthless, and obedient, which prevents them from seeking health care. As one widowed woman with a heart disease said: ‘My husband had a lot of assets, but I do not have anything now. I have no savings or property, so I am valueless for people’ (Hamiduzzaman et al. 2021c).

## Discussion

To our knowledge, this study is the first systematic review to provide an in-depth portrait of rural older adults’ whole health-seeking journey in rural areas of LMICs. We found that rural older adults in LMICs experience unique and multi-level challenges in seeking health care, such as inaccurate information and perceptions of health (individual level), the supportive or obstructive roles of family and social networks (interpersonal level), lack of access to formal healthcare facilities in rural areas (organization level), and multiple other social adversities (community and macrosystem level). More importantly, our findings also highlighted the resilience of older villagers: they creatively use informal institutions (e.g. alternative health care, personal connections) to navigate their healthcare-seeking journey, make sense of their health conditions, and ‘regain their worlds’ (Das et al. 2001, Lora-Wainwright 2013a).

Our review adds value to the existing literature on health-seeking behaviors through multidimensional and multilevel analyses using the social-ecological model (Poulin et al. 2020), which resonates with a large body of health services research worldwide (Phillips 1993, Kleinman et al. 2011, Lora-Wainwright 2013, Yang et al. 2014, Travers et al. 2020). In Congo, for example, Janzen found that people's decisions about health issues were inextricably linked to both the influence of other people (e.g. husbands, mothers-in-law) and what he called patients’ ‘therapy management groups’ (Janzen 1987). In another groundbreaking work of health-seeking, ‘*Logic of Care’* (2008), Mol suggested that the failure of diabetes management in The Netherlands was due to the adoption of the idealized logic of ‘individual choice’ and a lack of understanding of the complexity of the patient’s life experience (Mol 2008). Our findings are consistent with the perspectives of the aforementioned studies, which suggest that the HSB of rural older adults is best understood and interpreted within the socioecological framework of their own context, rather than through the assumptions of researchers and healthcare providers.

Our findings also enrich the literature on health system governance in LMICs. Formal institutions of healthcare, welfare, and other public goods often face significant resource constraints and structural challenges in LMICs (Tsai 2007). Our findings suggest that part of the reason rural older adults turn to informal systems for help is because of their unpleasant experiences with formal healthcare systems (Zanjani and Rowles 2012). Our findings also emphasize that the real interactions between institutions in rural areas of LMICs are more complex than the formal–informal dichotomy (Lauth 2000, Tsai 2006), and that the local informal health care solution is not inferior to the formal system (Shao 2016). In many cases, institutional change starts with informal coping strategies that local actors develop to circumvent the constraints of formal institutions (Lauth 2000). Because these informal coping strategies fit the local values and logic, they can then become a new formal institutional reality with repetition, diffusion, and endorsement by higher authorities (Tsai 2006), as seen in emerging examples within the health systems of rural Bolivia (Vandebroek et al. 2008), Nicaragua (Carrie et al. 2015), Nigeria (World Health Organization 2013), and Ghana (Jama et al. 2024). Therefore, it is important to consider how informal systems (e.g. alternative care) can inform formal systems in delivering contextually appropriate services to local populations (Tsai 2007).

This study has several limitations. We did not search for non-English articles or grey literature. As we did not include these studies, only five countries are represented in the included studies, which may have excluded important evidence from research-intensive LMICs (e.g. French-speaking Africa, Latin America) and limit the transferability of findings to other LMIC settings. In addition, the arbitrary classification of LMICs may cause confusion, as countries can shift categories over time with development (e.g. China). Another limitation is that although we tried to use inclusive criteria for original studies, we could only identify 19 studies with 484 participants (including caregivers) in our review. This situation confirms that older adults living in rural LMICs are still under-represented in the existing literature. Most of the included studies focused on the shortcomings of local health systems and overlooked innovative practices in rural LMICs, highlighting the need for more balanced research in the future (Harris et al. 2018). In addition, most of the qualitative studies in our review did not report detailed participant demographics (e.g. gender, education), making most subgroup analyses impossible. Despite these limitations, the review has several important strengths. First, although some of the identified themes were well documented in previous literature, this is the first review to organize multiple themes that emerged from the text through the socioecological framework and institutional adaptation model. The application of these frameworks provides a deeper understanding of older adults’ world. Second, we conducted an in-depth analysis of the methodological characteristics of the included studies, suggesting ways to improve future research. For example, our review identified only one phenomenological study and one ethnographic study, indicating that few studies have explored the essence of older adults’ health-seeking experiences or examined how they engage with rural health systems in their natural environments. This suggests a potential future research direction that warrants greater attention.

Our findings raise important issues for future practices, policy, and research. First, our findings might be useful for healthcare providers from LMICs. By examining the interactions between older adults and healthcare providers, our review underscored the influence of socioecological context in rural health practice (Travers et al. 2020, Golembiewski et al. 2022). It is important for providers to consider how local culture and structural inequalities shape patients’ HSBs and ability to navigate health systems, which also helps them to appreciate older adults’ wisdom and pursuit of wellbeing (Persson et al. 2019, Ma 2020). Second, in terms of health systems, our findings highlight the importance of informal institutions, such as alternative care providers, for older adults in rural LMICs. Care providers from informal institutions might be a resource for policymakers in their pursuit of a more equitable, culturally safe, and efficient health system to improve care for rural older adults in LMICs. Future policy analysis could explore the potential complementarity between informal and formal health systems in rural LMICs. This approach aligns with strategies such as the WHO Traditional Medicine Strategy 2025–2034 (World Health Organization 2025) and the Gujarat Declaration (WHO Traditional Complementary and Integrative Medicine Team 2023), which advocate for context-sensitive integration to advance UHC and health-related SDGs. Third, our findings highlight that healthcare systems in rural LMICs operate across multiple institutional layers, with different actors dominant in different contexts—formal institutions at the macro level, informal institutions at the interpersonal or local level. Recognizing these dynamics provides opportunities for system reform, e.g. policymakers can shape new institutional realities by leveraging informal coping strategies developed by local actors (Lauth 2000). Finally, as the population of rural older adults continues to grow, there is an urgent need for more research focused on this group. The paucity of studies in our review highlights a critical gap in the literature. In addition, more studies are needed that provide a balanced perspective on rural healthcare contexts, understand the resilience and agency in rural older adults’ health-seeking experiences, and offer constructive solutions to overcome identified barriers to care in rural LMICs.

## Conclusion

In this systematic review of rural older adults’ health-seeking experience in LMICs, we found that older adults had multi-level challenges during their health-seeking journeys. However, our analysis also showed that the older villagers in rural LMICs creatively used local informal institutions, such as alternative health care and personal connections, to help them navigate the rural health system when they needed health care. By highlighting both the unique challenges faced by this underrepresented population and the resilience and adaptive strategies they employ, our review advances understanding of patient agency in resource-limited settings. In addition, it identifies informal healthcare providers as an underutilized resource that could be strategically integrated into formal care systems to improve access for older populations. Future research should adopt a balanced perspective on rural healthcare contexts, understand the resilience and agency in rural older adults’ health-seeking experiences, and provide constructive solutions to overcome identified barriers to care in rural LMICs.

## Supplementary Material

czaf061_Supplementary_Data

## Data Availability

The full search strategy presented in this systematic review can be found in the online supplementary material. All the data used to generate the results are available to the public in the cited literature. The study protocol is available at https://www.crd.york.ac.uk/prospero/display_record.php?RecordID=358813.

## Author's note

^1^According to the World Bank's latest classification, the LMICs can be further divided into three groups based on their GDP per capita: (i) low-income countries, US $1145 or less; (ii) lower-middle-income countries, US $1146 to $4515; and (iii) upper-middle-income countries, US $4516 to $14 005. Monetary classifications mean that individual countries can change positions. For example, in this manuscript, while rural China was classified as an LMIC in our analysis, we acknowledge that, according to the World Bank's latest classification, China moved to an upper-middle-income category in 2019 (GDP per capita: US $12 614.1).

## Reflexivity statement

This research was conducted by a multidisciplinary team with members based in Canada, China, and Nigeria. The diversity of backgrounds and expertise allowed for a broad perspective on rural older people’s health-seeking experiences in low- and middle-income countries. The team also includes researchers at different career stages, from early-career scholars to senior academics. Early-career researchers played a central role in designing the study, conducting the analysis, and drafting the manuscript. We acknowledge that despite these strengths, challenges remain in fully representing the voices of all affected populations. The absence of authors based in low-income countries represents a limitation. To address this, the team presented the findings of this study at multiple international conferences (e.g. the Consortium of Universities for Global Health conference) and engaged with researchers and stakeholders from low-income settings to incorporate their perspectives. Their insights helped strengthen the analysis and improve the relevance of the findings.
