# Correction to: Older adults’ experiences of health seeking in rural areas in low- and middle-income countries: a systematic review of qualitative studies

**DOI:** 10.1093/heapol/czag102

**Published:** 2026-08-31

This is a correction to: Ziyue Wang, Xiaochen Ma, Can Su, Yihang Zhang, Xiang Zou, Mobolanle Balogun, Howard Bergman, Xiaoyun Liu, Nadia Sourial, Isabelle Vedel, Older adults’ experiences of health seeking in rural areas in low- and middle-income countries: a systematic review of qualitative studies, *Health Policy and Planning*, Volume 41, Issue 2, February 2026, Pages 299–313, https://doi.org/10.1093/heapol/czaf061

The following changes have been made to the originally published paper, erroneously maintained placeholder text throughout has been corrected with the reviewers’ details.

Text in section **Introduction, first paragraph, 2-4^th^ sentences,** are corrected to read as a footnote.

These emendation have been made to the paper.

