# Supplementary material for: Older adults’ experiences of health seeking in rural areas in low- and middle-income countries: a systematic review of qualitative studies
Source: Health Policy Plan. 2025 Oct 28;41(2):299–313. doi: 10.1093/heapol/czaf061 (PMC12906757; doi:10.1093/heapol/czaf061)
Supplement: czaf061_Supplementary_Data [file czaf061_supplementary_data.zip › Supplemental materials.docx]

Supplementary Materials to “Rural older people’s experiences of health-seeking in low- and middle-income countries (LMICs): a systematic review and thematic synthesis of qualitative studies”

Table of Content

[Supplemental Appendix 1. Full search strategy by database 3](#_Toc190116952)

[Supplemental Appendix 2: Hierarchical screening protocol and form used for review of full-text studies 13](#_Toc190116953)

[Supplemental Appendix 3: Data extraction protocol and form 14](#_Toc190116954)

[Supplemental Appendix 4. Detailed characteristics of 28 eligible articles 15](#_Toc190116955)

[Supplemental Appendix 5: Results of Mixed Methods Appraisal Tool (Green: ≥80, Yellow: 66-79, Red: <50) 25](#_Toc190116956)

[Supplemental Appendix 6: The social ecological model of health and Helmke and Levitsky’s (2004) institutional adaptation model 26](#_Toc190116957)

[References 28](#_Toc190116958)

# **Supplemental Appendix 1. Full search strategy by database**

**MEDLINE**

1. exp Qualitative Research/
2. (qualitative adj (research OR stud$3)).ti,ab,kf.
3. (mixed adj (method* OR studies)).ti,ab,kf.
4. exp Interviews as Topic/
5. interview*.ti,ab,kf.
6. focus groups/
7. focus group*.ti,ab,kf.
8. ((action OR participatory) and research).ti,ab,kf.
9. exp Community-Based Participatory Research/
10. grounded theory/
11. grounded theory.ti,ab,kf.
12. phenomenolog*.ti,ab,kf.
13. exp Narration/
14. narrat*.ti,ab,kf.
15. conversation*.ti,ab,kf.
16. discourse*.ti,ab,kf.
17. (ethnograph* OR ethnomethodolog* OR ethno methodolog* OR autoethnograph*).ti,ab,kf.
18. hermeneutic*.ti,ab,kf.
19. constructivis*.ti,ab,kf.
20. ((case OR field) adj (study OR studies)).ti,ab,kf.
21. ((participant* OR field) adj observ*).ti,ab,kf.
22. ((purpos* OR theoretical OR judg?ment OR "maximum variation" OR convenience OR "critical case" OR "deviant case" OR "key informant" OR snowball) adj sampl*).ti,ab,kf.
23. ((thematic OR content) adj analys*).ti,ab,kf.
24. "group discussion*".ti,ab,kf.
25. exp anthropology/ OR exp Anthropology, medical/ OR exp Anthropology, cultural/ OR anthropolog*.ti,ab,kf.
26. exp ethics/ OR exp ethics, professional/ OR exp Ethical Analysis/
27. OR/1-26
28. (afghanistan OR albania OR algeria OR american samoa OR angola OR "antigua and barbuda" OR antigua OR barbuda OR argentina OR armenia OR armenian OR aruba OR azerbaijan OR bangladesh OR barbados OR republic of belarus OR belarus OR byelarus OR belorussia OR byelorussian OR belize OR british honduras OR benin OR dahomey OR bhutan OR bolivia OR "bosnia and herzegovina" OR bosnia OR herzegovina OR botswana OR bechuanaland OR brazil OR brasil OR bulgaria OR burkina faso OR burkina fasso OR upper volta OR burundi OR urundi OR cabo verde OR cape verde OR cambodia OR kampuchea OR khmer republic OR cameroon OR cameron OR cameroun OR central african republic OR ubangi shari OR chad OR chile OR china OR colombia OR comoros OR comoro islands OR iles comers OR mayotte OR democratic republic of the congo OR democratic republic congo OR congo OR zaire OR costa rica OR "cote d’ivoire" OR "cote d’ ivoire" OR cote divoire OR cote d ivoire OR ivory coast OR croatia OR cuba OR cyprus OR djibouti OR french somaliland OR dominica OR dominican republic OR ecuador OR egypt OR united arab republic OR el salvador OR equatorial guinea OR spanish guinea OR eritrea OR eswatini OR swaziland OR ethiopia OR fiji OR gabon OR gabonese republic OR gambia OR "georgia (republic)" OR georgian OR ghana OR gold coast OR gibraltar OR greece OR grenada OR guam OR guatemala OR guinea OR guinea bissau OR guyana OR british guiana OR haiti OR hispaniola OR honduras OR hungary OR india OR indonesia OR timor OR iran OR iraq OR isle of man OR jamaica OR jordan OR kazakhstan OR kazakh OR kenya OR "democratic people’s republic of korea" OR north korea OR kosovo OR kyrgyzstan OR kirghizia OR kirgizstan OR kyrgyz republic OR kirghiz OR laos OR lao pdr OR "lao people's democratic republic" OR latvia OR lebanon OR lebanese republic OR lesotho OR basutoland OR liberia OR libya OR libyan arab jamahiriya OR republic of north macedonia OR macedonia OR madagascar OR malagasy republic OR malawi OR nyasaland OR malaysia OR malay federation OR malaya federation OR maldives OR indian ocean islands OR indian ocean OR mali OR malta OR micronesia OR federated states of micronesia OR kiribati OR marshall islands OR nauru OR northern mariana islands OR palau OR tuvalu OR mauritania OR mauritius OR mexico OR moldova OR moldovian OR mongolia OR montenegro OR morocco OR ifni OR mozambique OR portuguese east africa OR myanmar OR burma OR namibia OR nepal OR netherlands antilles OR nicaragua OR niger OR nigeria OR oman OR muscat OR pakistan OR panama OR papua new guinea OR new guinea OR paraguay OR peru OR philippines OR philipines OR phillipines OR phillippines OR romania OR russia OR russian federation OR ussr OR soviet union OR union of soviet socialist republics OR rwanda OR ruanda OR samoa OR pacific islands OR polynesia OR samoan islands OR navigator island OR navigator islands OR "sao tome and principe" OR saudi arabia OR senegal OR serbia OR seychelles OR sierra leone OR melanesia OR solomon island OR solomon islands OR norfolk island OR norfolk islands OR somalia OR south africa OR south sudan OR sri lanka OR ceylon OR "saint kitts and nevis" OR "st. kitts and nevis" OR saint lucia OR "st. lucia" OR "saint vincent and the grenadines" OR saint vincent OR "st. vincent" OR grenadines OR sudan OR suriname OR surinam OR dutch guiana OR netherlands guiana OR syria OR syrian arab republic OR tajikistan OR tadjikistan OR tadzhikistan OR tadzhik OR tanzania OR tanganyika OR thailand OR siam OR timor leste OR east timor OR togo OR togolese republic OR tonga OR "trinidad and tobago" OR trinidad OR tobago OR tunisia OR turkey OR turkmenistan OR turkmen OR uganda OR ukraine OR uruguay OR uzbekistan OR uzbek OR vanuatu OR new hebrides OR venezuela OR vietnam OR viet nam OR middle east OR west bank OR gaza OR palestine OR yemen OR yugoslavia OR zambia OR zimbabwe OR northern rhodesia OR global south OR africa south of the sahara OR sub-saharan africa OR subsaharan africa OR africa, central OR central africa OR africa, northern OR north africa OR northern africa OR magreb OR maghrib OR sahara OR africa, southern OR southern africa OR africa, eastern OR east africa OR eastern africa OR africa, western OR west africa OR western africa OR west indies OR indian ocean islands OR caribbean OR central america OR latin america OR "south and central america" OR south america OR asia, central OR central asia OR asia, northern OR north asia OR northern asia OR asia, southeastern OR southeastern asia OR south eastern asia OR southeast asia OR south east asia OR asia, western OR western asia OR europe, eastern OR east europe OR eastern europe OR developing country OR developing countries OR developing nation? OR developing population? OR developing world OR less developed countr* OR less developed nation? OR less developed population? OR less developed world OR lesser developed countr* OR lesser developed nation? OR lesser developed population? OR lesser developed world OR under developed countr* OR under developed nation? OR under developed population? OR under developed world OR underdeveloped countr* OR underdeveloped nation? OR underdeveloped population? OR underdeveloped world OR middle income countr* OR middle income nation? OR middle income population? OR low income countr* OR low income nation? OR low income population? OR lower income countr* OR lower income nation? OR lower income population? OR underserved countr* OR underserved nation? OR underserved population? OR underserved world OR under served countr* OR under served nation? OR under served population? OR under served world OR deprived countr* OR deprived nation? OR deprived population? OR deprived world OR poor countr* OR poor nation? OR poor population? OR poor world OR poorer countr* OR poorer nation? OR poorer population? OR poorer world OR developing econom* OR less developed econom* OR lesser developed econom* OR under developed econom* OR underdeveloped econom* OR middle income econom* OR low income econom* OR lower income econom* OR low gdp OR low gnp OR low gross domestic OR low gross national OR lower gdp OR lower gnp OR lower gross domestic OR lower gross national OR lmic OR lmics OR third world OR lami countr* OR transitional countr* OR emerging economies OR emerging nation?).ti,ab,sh,kf.
29. 25 and 26
30. Rural Health Services/ OR Rural Health/ OR Rural Nursing/ OR Medically Underserved Area/ OR Hospitals, Rural/ OR Rural Population/ OR Agricultural Workers' Diseases/ OR Farmers/ OR Agriculture/ OR rural*.mp. OR peasant*.mp. OR farmer*.mp. OR agricultural worker*.mp. OR sharecropp*.mp. OR ((remote OR nonmetropolitan OR non metropolitan OR developing OR less* developed OR under developed OR underdeveloped OR underserved OR under served OR deprived) adj3 (communit* OR area? OR village* OR region? OR setting*)).ti,ab,kf. OR ((remote OR nonmetropolitan OR non metropolitan OR underserved OR under served OR deprived OR village*) adj3 population*).ti,ab,kf. OR ((remote OR regional OR nonmetropolitan OR non metropolitan OR village*) adj3 (clinic? OR hospital? OR facility OR facilities OR health* center? OR health* centre? OR health care center? OR health care centre? OR medical center? OR medical centre?)).ti,ab,kf.
31. 27 and 28
32. exp aged/ OR aged worker/ OR ageism/ OR exp aging/ OR exp elderly care/ OR exp geriatrics/ OR (ag?ing OR elder* OR older* OR frail* OR ((aged OR old* OR senior) adj (adult* OR citizen* OR client* OR group* OR human* OR individual* OR inpatient* OR man OR men OR outpatient* OR patient* OR people OR person* OR population* OR resident* OR wom#n)) OR centenarian* OR "center* for the aged" OR "centre* for the aged" OR geriatric* OR gerontolog* OR long term care OR nonagenarian* OR octogenarian* OR old* age* OR retir* OR "senior center*" OR "senior centre*" OR seniors OR septuagenarian* OR sexagenarian* OR supercentenarian* OR (("69" OR "70" OR "74" OR "75" OR "79" OR "80" OR "84" OR "85" OR "89" OR "90" OR "94" OR "95") adj (aged OR year$1)) OR (("60" OR "65") adj2 (above OR older OR over OR plus))).mp.
33. 29 and 30
34. ((lived OR life OR personal* OR patient? OR survivor*) adj3 (experience* OR perspective* OR perception* OR meaning* OR view? OR viewpoint*)).ti,ab,kf.
35. exp "treatment adherence and compliance"/ OR health behavior/ OR health services accessibility/
36. exp Professional-Patient Relations/
37. Delayed Diagnosis/ OR Health Services Misuse/ OR Continuity of Patient Care/ OR "Health Knowledge, Attitudes, Practice"/ OR "Attitude of Health Personnel"/ OR Office Visits/
38. (((care OR diagnos* OR healthcare OR health OR treatment*) adj2 (access* OR seek*)) OR Screening OR (disease* adj test*) OR (Receiv* adj (diagnos* OR therap* OR treatment*)) OR (("Use" OR utili?ation) adj2 (care OR healthcare OR service*)) OR Patient follow-up OR Cope* OR Coping OR ((adher* OR Compliance OR concordan*) adj (drug* OR medication*)) OR ((adher* OR Compliance OR concordan*) adj3 (therap* OR treatment*)) OR Underus* OR "under us*").ti,ab,kf.
39. 32 OR 33 OR 34 OR 35 OR 36
40. 31 and 37
41. limit 38 to yr="2002 -Current"
42. (cross-sectional stud* OR case-control stud* OR cohort stud* OR panel OR modelling OR multilevel analys?s OR cost-effectiveness OR cost-utility OR cost-benefit OR Cost Analysis OR economic evaluation*).ti.
43. (guideline* OR practice guideline*).pt. OR exp guidelines as topic/ OR review*.pt. OR review literature as topic/ OR systematic review*.pt. OR systematic reviews as topic/ OR comment*.pt. OR editorial*.pt. OR meta-analys?s.pt. OR exp meta-analysis as topic/ OR systematic review*.ti. OR literature review*.ti. OR meta analys?s.ti. OR scoping review*.ti. OR rapid review*.ti.
44. 40 OR 41
45. 39 not 42
46. limit 44 to (english language and humans)

**Embase**

1. exp qualitative research/
2. (qualitative ADJ (research OR stud$3)).ti,ab,kf.
3. (mixed ADJ (method* OR studies)).ti,ab,kf.
4. exp interview/
5. 4 NOT (exp Delphi study/)
6. interview*.ti,ab,kf.
7. focus group*.ti,ab,kf.
8. ((action OR participatory) and research).ti,ab,kf.
9. exp participatory research/
10. grounded theory/
11. grounded theory.ti,ab,kf.
12. phenomenolog*.ti,ab,kf.
13. exp narrative/
14. narrat*.ti,ab,kf.
15. conversation*.ti,ab,kf.
16. discourse*.ti,ab,kf.
17. (ethnograph* OR ethnomethodolog* OR ethno methodolog* OR autoethnograph*).ti,ab,kf.
18. hermeneutic*.ti,ab,kf.
19. constructivis*.ti,ab,kf.
20. ((case*) ADJ (study OR studies)).ti,ab,kf.
21. ((participant* OR field) ADJ observ*).ti,ab,kf.
22. ((purpos* OR theoretical OR judg?ment OR maximum variation OR convenience OR critical case OR deviant case OR key informant OR snowball) ADJ sampl*).ti,ab,kf.
23. ((thematic OR content) ADJ analys*).ti,ab,kf.
24. group discussion*.ti,ab,kf.
25. exp anthropology/ OR exp medical anthropology/ OR exp cultural anthropology/ OR (anthropolog*).ti,ab,kf.
26. exp ethics/
27. 1 OR 2 OR 3 OR 5 OR 6 OR 7 OR 8 OR 9 OR 10 OR 11 OR 12 OR 13 OR 14 OR 15 OR 16 OR 17 OR 18 OR 19 OR 20 OR 21 OR 22 OR 23 OR 24 OR 25 OR 26
28. (afghanistan OR albania OR algeria OR american samoa OR angola OR "antigua and barbuda" OR antigua OR barbuda OR argentina OR armenia OR armenian OR aruba OR azerbaijan OR bangladesh OR barbados OR republic of belarus OR belarus OR byelarus OR belorussia OR byelorussian OR belize OR british honduras OR benin OR dahomey OR bhutan OR bolivia OR "bosnia and herzegovina" OR bosnia OR herzegovina OR botswana OR bechuanaland OR brazil OR brasil OR bulgaria OR burkina faso OR burkina fasso OR upper volta OR burundi OR urundi OR cabo verde OR cape verde OR cambodia OR kampuchea OR khmer republic OR cameroon OR cameron OR cameroun OR central african republic OR ubangi shari OR chad OR chile OR china OR colombia OR comoros OR comoro islands OR iles comores OR mayotte OR democratic republic of the congo OR democratic republic congo OR congo OR zaire OR costa rica OR "cote d’ivoire" OR "cote d’ ivoire" OR cote divoire OR cote d ivoire OR ivory coast OR croatia OR cuba OR cyprus OR djibouti OR french somaliland OR dominica OR dominican republic OR ecuador OR egypt OR united arab republic OR el salvador OR equatorial guinea OR spanish guinea OR eritrea OR eswatini OR swaziland OR ethiopia OR fiji OR gabon OR gabonese republic OR gambia OR "georgia (republic)" OR georgian OR ghana OR gold coast OR gibraltar OR greece OR grenada OR guam OR guatemala OR guinea OR guinea bissau OR guyana OR british guiana OR haiti OR hispaniola OR honduras OR hungary OR india OR indonesia OR timor OR iran OR iraq OR isle of man OR jamaica OR jordan OR kazakhstan OR kazakh OR kenya OR "democratic people’s republic of korea" OR north korea OR kosovo OR kyrgyzstan OR kirghizia OR kirgizstan OR kyrgyz republic OR kirghiz OR laos OR lao pdr OR "lao people's democratic republic" OR latvia OR lebanon OR lebanese republic OR lesotho OR basutoland OR liberia OR libya OR libyan arab jamahiriya OR republic of north macedonia OR macedonia OR madagascar OR malagasy republic OR malawi OR nyasaland OR malaysia OR malay federation OR malaya federation OR maldives OR indian ocean islands OR indian ocean OR mali OR malta OR micronesia OR federated states of micronesia OR kiribati OR marshall islands OR nauru OR northern mariana islands OR palau OR tuvalu OR mauritania OR mauritius OR mexico OR moldova OR moldovian OR mongolia OR montenegro OR "montenegro (republic)" OR morocco OR ifni OR mozambique OR portuguese east africa OR myanmar OR burma OR namibia OR nepal OR netherlands antilles OR nicaragua OR niger OR nigeria OR oman OR muscat OR pakistan OR panama OR papua new guinea OR new guinea OR paraguay OR peru OR philippines OR philipines OR phillipines OR phillippines OR romania OR russia OR russian federation OR ussr OR soviet union OR union of soviet socialist republics OR rwanda OR ruanda OR samoa OR pacific islands OR polynesia OR samoan islands OR navigatOR island OR navigatOR islands OR "sao tome and principe" OR saudi arabia OR senegal OR serbia OR seychelles OR sierra leone OR melanesia OR solomon island OR solomon islands OR norfolk island OR norfolk islands OR somalia OR south africa OR south sudan OR sri lanka OR ceylon OR "saint kitts and nevis" OR "st. kitts and nevis" OR saint lucia OR "st. lucia" OR "saint vincent and the grenadines" OR saint vincent OR "st. vincent" OR grenadines OR sudan OR suriname OR surinam OR dutch guiana OR netherlands guiana OR syria OR syrian arab republic OR tajikistan OR tadjikistan OR tadzhikistan OR tadzhik OR tanzania OR tanganyika OR thailand OR siam OR timor leste OR east timor OR togo OR togolese republic OR tonga OR "trinidad and tobago" OR trinidad OR tobago OR tunisia OR "turkey (republic)" OR turkey OR turkmenistan OR turkmen OR uganda OR ukraine OR uruguay OR uzbekistan OR uzbek OR vanuatu OR new hebrides OR venezuela OR vietnam OR viet nam OR middle east OR west bank OR gaza OR palestine OR yemen OR yugoslavia OR zambia OR zimbabwe OR northern rhodesia OR global south OR africa south of the sahara OR "sub saharan africa" OR subsaharan africa OR africa, central OR central africa OR africa, northern OR north africa OR northern africa OR magreb OR maghrib OR sahara OR africa, southern OR southern africa OR africa, eastern OR east africa OR eastern africa OR africa, western OR west africa OR western africa OR west indies OR indian ocean islands OR caribbean region OR caribbean islands OR caribbean OR central america OR latin america OR "south and central america" OR south america OR asia, central OR central asia OR asia, northern OR north asia OR northern asia OR asia, southeastern OR southeastern asia OR south eastern asia OR southeast asia OR south east asia OR asia, western OR western asia OR europe, eastern OR east europe OR eastern europe OR developing country OR developing countries OR developing nation? OR developing population? OR developing world OR less developed countr* OR less developed nation? OR less developed population? OR less developed world OR lesser developed countr* OR lesser developed nation? OR lesser developed population? OR lesser developed world OR under developed countr* OR under developed nation? OR under developed population? OR under developed world OR underdeveloped countr* OR underdeveloped nation? OR underdeveloped population? OR underdeveloped world OR middle income countr* OR middle income nation? OR middle income population? OR low income countr* OR low income nation? OR low income population? OR lower income countr* OR lower income nation? OR lower income population? OR underserved countr* OR underserved nation? OR underserved population? OR underserved world OR under served countr* OR under served nation? OR under served population? OR under served world OR deprived countr* OR deprived nation? OR deprived population? OR deprived world OR poor countr* OR poor nation? OR poor population? OR poor world OR poorer countr* OR poorer nation? OR poorer population? OR poorer world OR developing econom* OR less developed econom* OR lesser developed econom* OR under developed econom* OR underdeveloped econom* OR middle income econom* OR low income econom* OR lower income econom* OR low gdp OR low gnp OR low gross domestic OR low gross national OR lower gdp OR lower gnp OR lower gross domestic OR lower gross national OR lmic OR lmics OR third world OR lami countr* OR transitional countr* OR emerging economies OR emerging nation?).ti,ab,sh,kw.
29. 25 AND 26
30. rural health care/ OR rural health nursing/ OR rural health/ OR rural hospital/ OR rural population/ OR agriculture/ OR agricultural worker/ OR rural*.mp. OR agricultural worker*.mp. OR peasant*.mp. OR farmer*.mp. OR sharecropp*.mp. OR ((remote OR nonmetropolitan OR non metropolitan OR developing OR less* developed OR under developed OR underdeveloped OR underserved OR under served OR deprived OR poor) ADJ3 (communit* OR area? OR village* OR region? OR setting*)).ti,ab,kf. OR ((remote OR nonmetropolitan OR non metropolitan OR underserved OR under served OR deprived OR poor OR village*) ADJ3 population*).ti,ab,kf. OR ((remote OR regional OR nonmetropolitan OR non metropolitan OR village*) ADJ3 (clinic? OR hospital? OR facility OR facilities OR health* center? OR health* centre? OR health care center? OR health care centre? OR medical center? OR medical centre?)).ti,ab,kf.
31. 27 AND 28
32. exp aged/ OR exp aged worker/ OR exp ageism/ OR exp aging/ OR exp healthy aging OR exp elderly care/ OR exp geriatrics/ OR (ag?ing OR elder* OR frail* OR ((aged OR old* OR senior) ADJ (adult* OR citizen* OR client* OR group* OR human* OR individual* OR inpatient* OR man OR men OR outpatient* OR patient* OR people OR person* OR population* OR resident* OR wom#n)) OR centenarian* OR center* for the aged OR centre* for the aged OR geriatric* OR gerontolog* OR long term care OR nonagenarian* OR octogenarian* OR old* age* OR retir* OR senior center* OR senior centre* OR seniors OR septuagenarian* OR sexagenarian* OR supercentenarian* OR ((69 OR 70 OR 74 OR 75 OR 79 OR 80 OR 84 OR 85 OR 89 OR 90 OR 94 OR 95) ADJ (aged OR year$1)) OR ((60 OR 65) ADJ2 (above OR older OR over OR plus))).mp.
33. 29 AND 30
34. ((lived OR life OR personal* OR patient? OR survivor*) ADJ3 (experience* OR perspective* OR perception* OR meaning* OR view? OR viewpoint*)).ti,ab,kf.
35. exp patient compliance/ OR health behavior/ OR health care access/
36. exp professional-patient relationship/
37. delayed diagnosis/ OR holistic care/ OR transitional care/ OR attitude to health/ OR health personnel attitude/ OR patient attitude/ OR patient decision making/ OR ambulatory care/
38. (((care OR diagnos* OR healthcare OR health OR treatment*) ADJ2 (access* OR seek*)) OR Screening OR (disease* ADJ test*) OR (Receiv* ADJ (diagnos* OR therap* OR treatment*)) OR (("Use" OR utilization OR utilisation) ADJ2 (care OR healthcare OR service*)) OR Patient follow-up OR Cope* OR Coping OR ((adher* OR Compliance OR concordan*) ADJ (drug* OR medication*)) OR ((adher* OR Compliance OR concordan*) ADJ3 (therap* OR treatment*)) OR Underus* OR under us*).ti,ab,kf.
39. 32 OR 33 OR 34 OR 35 OR 36
40. 31 AND 37
41. limit 38 to yr="2002"
42. (cross-sectional stud* OR case-control stud* OR cohort stud* OR panel OR modelling OR multilevel analys?s OR cost-effectiveness OR cost-utility OR cost-benefit OR Cost Analysis OR economic evaluation*).ti.
43. (guideline* OR practice guideline*).pt. OR exp practice guideline/ OR review*.pt. OR review/ OR systematic review*.pt. OR comment*.pt. OR editorial*.pt. OR meta-analys?s.pt. OR exp meta-analys?s/ OR systematic review*.ti. OR literature review*.ti. OR meta analys?s.ti. OR scoping review*.ti. OR rapid review*.ti.
44. 40 OR 41
45. 39 NOT 42
46. limit 43 to (human and english language)

**CINAHL**

1. (MH " Qualitative Studies+")
2. ((TI qualitative OR AB qualitative) W1 ((TI research OR AB research) OR (TI stud*3 OR AB stud*3)))
3. ((TI mixed OR AB mixed) W1 ((TI method* OR AB method*) OR (TI studies OR AB studies)))
4. (MH " Interviews+")
5. (TI interview* OR AB interview*)
6. (MH "focus groups")
7. (TI "focus group*" OR AB "focus group*")
8. (((TI action OR AB action) OR (TI participatory OR AB participatory)) AND (TI research OR AB research))
9. (MH "Action Research+")
10. (MH "grounded theory")
11. (TI "grounded theory" OR AB "grounded theory")
12. (TI phenomenolog* OR AB phenomenolog*) OR (MH " phenomenology") OR (MH "phenomenology Research")
13. (MH "Narratives+")
14. (MH "Observational Methods+")
15. (TI narrat* OR AB narrat*)
16. (TI conversation* OR AB conversation*) OR (MH "conversation")
17. (TI discourse* OR AB discourse*) OR (MH " discourse analysis")
18. ((TI ethnograph* OR AB ethnograph*) OR (TI ethnomethodolog* OR AB ethnomethodolog*) OR (TI "ethno methodolog*" OR AB "ethno methodolog*") OR (TI autoethnograph* OR AB autoethnograph*))
19. (TI hermeneutic* OR AB hermeneutic*)
20. (TI constructivis* OR AB constructivis*) OR (MH "social constructivism")
21. (((TI case OR AB case) W1 ((TI study OR AB study) OR (TI studies OR AB studies)))
22. (((TI participant* OR AB participant*) OR (TI field OR AB field)) W1 (TI observ* OR AB observ*))
23. (((TI purpos* OR AB purpos*) OR (TI theoretical OR AB theoretical) OR (TI judg#ment OR AB judg#ment) OR (TI "maximum variation" OR AB "maximum variation") OR (TI convenience OR AB convenience) OR (TI "critical case" OR AB "critical case") OR (TI "deviant case" OR AB "deviant case") OR (TI "key informant" OR AB "key informant") OR (TI snowball OR AB snowball) W1 (TI sampl* OR AB sampl*))
24. (((TI thematic OR AB thematic) OR (TI content OR AB content)) W1 (TI analys* OR AB analys*))
25. (TI "group discussion*" OR AB "group discussion*")
26. (MH "Anthropology”) OR (MH "Anthropology, cultural") OR (TI Anthropolog* OR AB Anthropolog*)
27. 1 OR 2 OR 3 OR 4 OR 5 OR 6 OR 7 OR 8 OR 9 OR 10 OR 11 OR 12 OR 13 OR 14 OR 15 OR 16 OR 17 OR 18 OR 19 OR 20 OR 21 OR 22 OR 23 OR 24 OR 25 OR 26 OR 27
28. ((TI afghanistan OR AB afghanistan) OR (TI albania OR AB albania) OR (TI algeria OR AB algeria) OR (TI "american samoa" OR AB "american samoa") OR (TI angola OR AB angola) OR (TI "antigua and barbuda" OR AB "antigua and barbuda") OR (TI antigua OR AB antigua) OR (TI barbuda OR AB barbuda) OR (TI argentina OR AB argentina) OR (TI armenia OR AB armenia) OR (TI armenian OR AB armenian) OR (TI aruba OR AB aruba) OR (TI azerbaijan OR AB azerbaijan) OR (TI bahrain OR AB bahrain) OR (TI bangladesh OR AB bangladesh) OR (TI barbados OR AB barbados) OR (TI "republic of belarus" OR AB "republic of belarus") OR (TI belarus OR AB belarus) OR (TI byelarus OR AB byelarus) OR (TI belorussia OR AB belorussia) OR (TI byelorussian OR AB byelorussian) OR (TI belize OR AB belize) OR (TI "british honduras" OR AB "british honduras") OR (TI benin OR AB benin) OR (TI dahomey OR AB dahomey) OR (TI bhutan OR AB bhutan) OR (TI bolivia OR AB bolivia) OR (TI "bosnia and herzegovina" OR AB "bosnia and herzegovina") OR (TI bosnia OR AB bosnia) OR (TI herzegovina OR AB herzegovina) OR (TI botswana OR AB botswana) OR (TI bechuanaland OR AB bechuanaland) OR (TI brazil OR AB brazil) OR (TI brasil OR AB brasil) OR (TI bulgaria OR AB bulgaria) OR (TI "burkina faso" OR AB "burkina faso") OR (TI "burkina fasso" OR AB "burkina fasso") OR (TI "upper volta" OR AB "upper volta") OR (TI burundi OR AB burundi) OR (TI urundi OR AB urundi) OR (TI "cabo verde" OR AB "cabo verde") OR (TI "cape verde" OR AB "cape verde") OR (TI cambodia OR AB cambodia) OR (TI kampuchea OR AB kampuchea) OR (TI "khmer republic" OR AB "khmer republic") OR (TI cameroon OR AB cameroon) OR (TI cameron OR AB cameron) OR (TI cameroun OR AB cameroun) OR (TI "central african republic" OR AB "central african republic") OR (TI "ubangi shari" OR AB "ubangi shari") OR (TI chad OR AB chad) OR (TI chile OR AB chile) OR (TI china OR AB china) OR (TI colombia OR AB colombia) OR (TI comoros OR AB comoros) OR (TI "comoro islands" OR AB "comoro islands") OR (TI "iles comers" OR AB "iles comers") OR (TI mayotte OR AB mayotte) OR (TI "democratic republic of the congo" OR AB "democratic republic of the congo") OR (TI "democratic republic congo" OR AB "democratic republic congo") OR (TI congo OR AB congo) OR (TI zaire OR AB zaire) OR (TI "costa rica" OR AB "costa rica") OR (TI "cote d’ivoire" OR AB "cote d’ivoire") OR (TI "cote d’ ivoire" OR AB "cote d’ ivoire") OR (TI "cote divoire" OR AB "cote divoire") OR (TI "cote d ivoire" OR AB "cote d ivoire") OR (TI "ivory coast" OR AB "ivory coast") OR (TI croatia OR AB croatia) OR (TI cuba OR AB cuba) OR (TI cyprus OR AB cyprus) OR (TI djibouti OR AB djibouti) OR (TI "french somaliland" OR AB "french somaliland") OR (TI dominica OR AB dominica) OR (TI "dominican republic" OR AB "dominican republic") OR (TI ecuador OR AB ecuador) OR (TI egypt OR AB egypt) OR (TI "united arab republic" OR AB "united arab republic") OR (TI "el salvador" OR AB "el salvador") OR (TI "equatorial guinea" OR AB "equatorial guinea") OR (TI "spanish guinea" OR AB "spanish guinea") OR (TI eritrea OR AB eritrea) OR (TI eswatini OR AB eswatini) OR (TI swaziland OR AB swaziland) OR (TI ethiopia OR AB ethiopia) OR (TI fiji OR AB fiji) OR (TI gabon OR AB gabon) OR (TI "gabonese republic" OR AB "gabonese republic") OR (TI gambia OR AB gambia) OR (TI "georgia (republic)" OR AB "georgia (republic)") OR (TI georgian OR AB georgian) OR (TI ghana OR AB ghana) OR (TI "gold coast" OR AB "gold coast") OR (TI gibraltar OR AB gibraltar) OR (TI greece OR AB greece) OR (TI grenada OR AB grenada) OR (TI guam OR AB guam) OR (TI guatemala OR AB guatemala) OR (TI guinea OR AB guinea) OR (TI "guinea bissau" OR AB "guinea bissau") OR (TI guyana OR AB guyana) OR (TI "british guiana" OR AB "british guiana") OR (TI haiti OR AB haiti) OR (TI hispaniola OR AB hispaniola) OR (TI honduras OR AB honduras) OR (TI hungary OR AB hungary) OR (TI india OR AB india) OR (TI indonesia OR AB indonesia) OR (TI timor OR AB timor) OR (TI iran OR AB iran) OR (TI iraq OR AB iraq) OR (TI "isle of man" OR AB "isle of man") OR (TI jamaica OR AB jamaica) OR (TI jordan OR AB jordan) OR (TI kazakhstan OR AB kazakhstan) OR (TI kazakh OR AB kazakh) OR (TI kenya OR AB kenya) OR (TI "democratic people’s republic of korea" OR AB "democratic people’s republic of korea") OR (TI "north korea" OR AB "north korea") OR (TI kosovo OR AB kosovo) OR (TI kyrgyzstan OR AB kyrgyzstan) OR (TI kirghizia OR AB kirghizia) OR (TI kirgizstan OR AB kirgizstan) OR (TI "kyrgyz republic" OR AB "kyrgyz republic") OR (TI kirghiz OR AB kirghiz) OR (TI laos OR AB laos) OR (TI "lao pdr" OR AB "lao pdr") OR (TI "lao people's democratic republic" OR AB "lao people's democratic republic") OR (TI latvia OR AB latvia) OR (TI lebanon OR AB lebanon) OR (TI "lebanese republic" OR AB "lebanese republic") OR (TI lesotho OR AB lesotho) OR (TI basutoland OR AB basutoland) OR (TI liberia OR AB liberia) OR (TI libya OR AB libya) OR (TI "libyan arab jamahiriya" OR AB "libyan arab jamahiriya") OR (TI "republic of north macedonia" OR AB "republic of north macedonia") OR (TI macedonia OR AB macedonia) OR (TI madagascar OR AB madagascar) OR (TI "malagasy republic" OR AB "malagasy republic") OR (TI malawi OR AB malawi) OR (TI nyasaland OR AB nyasaland) OR (TI malaysia OR AB malaysia) OR (TI "malay federation" OR AB "malay federation") OR (TI "malaya federation" OR AB "malaya federation") OR (TI maldives OR AB maldives) OR (TI "indian ocean islands" OR AB "indian ocean islands") OR (TI "indian ocean" OR AB "indian ocean") OR (TI mali OR AB mali) OR (TI malta OR AB malta) OR (TI micronesia OR AB micronesia) OR (TI "federated states of micronesia" OR AB "federated states of micronesia") OR (TI kiribati OR AB kiribati) OR (TI "marshall islands" OR AB "marshall islands") OR (TI nauru OR AB nauru) OR (TI "northern mariana islands" OR AB "northern mariana islands") OR (TI palau OR AB palau) OR (TI tuvalu OR AB tuvalu) OR (TI mauritania OR AB mauritania) OR (TI mauritius OR AB mauritius) OR (TI mexico OR AB mexico) OR (TI moldova OR AB moldova) OR (TI moldovian OR AB moldovian) OR (TI mongolia OR AB mongolia) OR (TI montenegro OR AB montenegro) OR (TI morocco OR AB morocco) OR (TI ifni OR AB ifni) OR (TI mozambique OR AB mozambique) OR (TI "portuguese east africa" OR AB "portuguese east africa") OR (TI myanmar OR AB myanmar) OR (TI burma OR AB burma) OR (TI namibia OR AB namibia) OR (TI nepal OR AB nepal) OR (TI "netherlands antilles" OR AB "netherlands antilles") OR (TI nicaragua OR AB nicaragua) OR (TI niger OR AB niger) OR (TI nigeria OR AB nigeria) OR (TI oman OR AB oman) OR (TI muscat OR AB muscat) OR (TI pakistan OR AB pakistan) OR (TI panama OR AB panama) OR (TI "papua new guinea" OR AB "papua new guinea") OR (TI "new guinea" OR AB "new guinea") OR (TI paraguay OR AB paraguay) OR (TI peru OR AB peru) OR (TI philippines OR AB philippines) OR (TI philipines OR AB philipines) OR (TI phillipines OR AB phillipines) OR (TI phillippines OR AB phillippines) OR (TI romania OR AB romania) OR (TI russia OR AB russia) OR (TI "russian federation" OR AB "russian federation") OR (TI ussr OR AB ussr) OR (TI "soviet union" OR AB "soviet union") OR (TI "union of soviet socialist republics" OR AB "union of soviet socialist republics") OR (TI rwanda OR AB rwanda) OR (TI ruanda OR AB ruanda) OR (TI samoa OR AB samoa) OR (TI "pacific islands" OR AB "pacific islands") OR (TI polynesia OR AB polynesia) OR (TI "samoan islands" OR AB "samoan islands") OR (TI "navigator island" OR AB "navigator island") OR (TI "navigator islands" OR AB "navigator islands") OR (TI "sao tome and principe" OR AB "sao tome and principe") OR (TI "saudi arabia" OR AB "saudi arabia") OR (TI senegal OR AB senegal) OR (TI serbia OR AB serbia) OR (TI seychelles OR AB seychelles) OR (TI "sierra leone" OR AB "sierra leone") OR (TI melanesia OR AB melanesia) OR (TI "solomon island" OR AB "solomon island") OR (TI "solomon islands" OR AB "solomon islands") OR (TI "norfolk island" OR AB "norfolk island") OR (TI "norfolk islands" OR AB "norfolk islands") OR (TI somalia OR AB somalia) OR (TI "south africa" OR AB "south africa") OR (TI "south sudan" OR AB "south sudan") OR (TI "sri lanka" OR AB "sri lanka") OR (TI ceylon OR AB ceylon) OR (TI "saint kitts and nevis" OR AB "saint kitts and nevis") OR (TI "st. kitts and nevis" OR AB "st. kitts and nevis") OR (TI "saint lucia" OR AB "saint lucia") OR (TI "st. lucia" OR AB "st. lucia") OR (TI "saint vincent and the grenadines" OR AB "saint vincent and the grenadines") OR (TI "saint vincent" OR AB "saint vincent") OR (TI "st. vincent" OR AB "st. vincent") OR (TI grenadines OR AB grenadines) OR (TI sudan OR AB sudan) OR (TI suriname OR AB suriname) OR (TI surinam OR AB surinam) OR (TI "dutch guiana" OR AB "dutch guiana") OR (TI "netherlands guiana" OR AB "netherlands guiana") OR (TI syria OR AB syria) OR (TI "syrian arab republic" OR AB "syrian arab republic") OR (TI tajikistan OR AB tajikistan) OR (TI tadjikistan OR AB tadjikistan) OR (TI tadzhikistan OR AB tadzhikistan) OR (TI tadzhik OR AB tadzhik) OR (TI tanzania OR AB tanzania) OR (TI tanganyika OR AB tanganyika) OR (TI thailand OR AB thailand) OR (TI siam OR AB siam) OR (TI "timor leste" OR AB "timor leste") OR (TI "east timor" OR AB "east timor") OR (TI togo OR AB togo) OR (TI "togolese republic" OR AB "togolese republic") OR (TI tonga OR AB tonga) OR (TI "trinidad and tobago" OR AB "trinidad and tobago") OR (TI trinidad OR AB trinidad) OR (TI tobago OR AB tobago) OR (TI tunisia OR AB tunisia) OR (TI turkey OR AB turkey) OR (TI turkmenistan OR AB turkmenistan) OR (TI turkmen OR AB turkmen) OR (TI uganda OR AB uganda) OR (TI ukraine OR AB ukraine) OR (TI uruguay OR AB uruguay) OR (TI uzbekistan OR AB uzbekistan) OR (TI uzbek OR AB uzbek) OR (TI vanuatu OR AB vanuatu) OR (TI "new hebrides" OR AB "new hebrides") OR (TI venezuela OR AB venezuela) OR (TI vietnam OR AB vietnam) OR (TI "viet nam" OR AB "viet nam") OR (TI "middle east" OR AB "middle east") OR (TI "west bank" OR AB "west bank") OR (TI gaza OR AB gaza) OR (TI palestine OR AB palestine) OR (TI yemen OR AB yemen) OR (TI yugoslavia OR AB yugoslavia) OR (TI zambia OR AB zambia) OR (TI zimbabwe OR AB zimbabwe) OR (TI "northern rhodesia" OR AB "northern rhodesia") OR (TI "global south" OR AB "global south") OR (TI "africa south of the sahara" OR AB "africa south of the sahara") OR (TI "sub-saharan africa" OR AB "sub-saharan africa") OR (TI "subsaharan africa" OR AB "subsaharan africa") OR (TI "africa, central" OR AB "africa, central") OR (TI "central africa" OR AB "central africa") OR (TI "africa, northern" OR AB "africa, northern") OR (TI "north africa" OR AB "north africa") OR (TI "northern africa" OR AB "northern africa") OR (TI magreb OR AB magreb) OR (TI maghrib OR AB maghrib) OR (TI sahara OR AB sahara) OR (TI "africa, southern" OR AB "africa, southern") OR (TI "southern africa" OR AB "southern africa") OR (TI "africa, eastern" OR AB "africa, eastern") OR (TI "east africa" OR AB "east africa") OR (TI "eastern africa" OR AB "eastern africa") OR (TI "africa, western" OR AB "africa, western") OR (TI "west africa" OR AB "west africa") OR (TI "western africa" OR AB "western africa") OR (TI "west indies" OR AB "west indies") OR (TI "indian ocean islands" OR AB "indian ocean islands") OR (TI caribbean OR AB caribbean) OR (TI "central america" OR AB "central america") OR (TI "latin america" OR AB "latin america") OR (TI "south and central america" OR AB "south and central america") OR (TI "south america" OR AB "south america") OR (TI "asia, central" OR AB "asia, central") OR (TI "central asia" OR AB "central asia") OR (TI "asia, northern" OR AB "asia, northern") OR (TI "north asia" OR AB "north asia") OR (TI "northern asia" OR AB "northern asia") OR (TI "asia, southeastern" OR AB "asia, southeastern") OR (TI "southeastern asia" OR AB "southeastern asia") OR (TI "south eastern asia" OR AB "south eastern asia") OR (TI "southeast asia" OR AB "southeast asia") OR (TI "south east asia" OR AB "south east asia") OR (TI "asia, western" OR AB "asia, western") OR (TI "western asia" OR AB "western asia") OR (TI "europe, eastern" OR AB "europe, eastern") OR (TI "east europe" OR AB "east europe") OR (TI "eastern europe" OR AB "eastern europe") OR (TI "developing country" OR AB "developing country") OR (TI "developing countries" OR AB "developing countries") OR (TI "developing nation#" OR AB "developing nation#") OR (TI "developing population#" OR AB "developing population#") OR (TI "developing world" OR AB "developing world") OR (TI "less developed countr*" OR AB "less developed countr*") OR (TI "less developed nation#" OR AB "less developed nation#") OR (TI "less developed population#" OR AB "less developed population#") OR (TI "less developed world" OR AB "less developed world") OR (TI "lesser developed countr*" OR AB "lesser developed countr*") OR (TI "lesser developed nation#" OR AB "lesser developed nation#") OR (TI "lesser developed population#" OR AB "lesser developed population#") OR (TI "lesser developed world" OR AB "lesser developed world") OR (TI "under developed countr*" OR AB "under developed countr*") OR (TI "under developed nation#" OR AB "under developed nation#") OR (TI "under developed population#" OR AB "under developed population#") OR (TI "under developed world" OR AB "under developed world") OR (TI "underdeveloped countr*" OR AB "underdeveloped countr*") OR (TI "underdeveloped nation#" OR AB "underdeveloped nation#") OR (TI "underdeveloped population#" OR AB "underdeveloped population#") OR (TI "underdeveloped world" OR AB "underdeveloped world") OR (TI "middle income countr*" OR AB "middle income countr*") OR (TI "middle income nation#" OR AB "middle income nation#") OR (TI "middle income population#" OR AB "middle income population#") OR (TI "low income countr*" OR AB "low income countr*") OR (TI "low income nation#" OR AB "low income nation#") OR (TI "low income population#" OR AB "low income population#") OR (TI "lower income countr*" OR AB "lower income countr*") OR (TI "lower income nation#" OR AB "lower income nation#") OR (TI "lower income population#" OR AB "lower income population#") OR (TI "underserved countr*" OR AB "underserved countr*") OR (TI "underserved nation#" OR AB "underserved nation#") OR (TI "underserved population#" OR AB "underserved population#") OR (TI "underserved world" OR AB "underserved world") OR (TI "under served countr*" OR AB "under served countr*") OR (TI "under served nation#" OR AB "under served nation#") OR (TI "under served population#" OR AB "under served population#") OR (TI "under served world" OR AB "under served world") OR (TI "deprived countr*" OR AB "deprived countr*") OR (TI "deprived nation#" OR AB "deprived nation#") OR (TI "deprived population#" OR AB "deprived population#") OR (TI "deprived world" OR AB "deprived world") OR (TI "poor countr*" OR AB "poor countr*") OR (TI "poor nation#" OR AB "poor nation#") OR (TI "poor population#" OR AB "poor population#") OR (TI "poor world" OR AB "poor world") OR (TI "poorer countr*" OR AB "poorer countr*") OR (TI "poorer nation#" OR AB "poorer nation#") OR (TI "poorer population#" OR AB "poorer population#") OR (TI "poorer world" OR AB "poorer world") OR (TI "developing econom*" OR AB "developing econom*") OR (TI "less developed econom*" OR AB "less developed econom*") OR (TI "lesser developed econom*" OR AB "lesser developed econom*") OR (TI "under developed econom*" OR AB "under developed econom*") OR (TI "underdeveloped econom*" OR AB "underdeveloped econom*") OR (TI "middle income econom*" OR AB "middle income econom*") OR (TI "low income econom*" OR AB "low income econom*") OR (TI "lower income econom*" OR AB "lower income econom*") OR (TI "low gdp" OR AB "low gdp") OR (TI "low gnp" OR AB "low gnp") OR (TI "low gross domestic" OR AB "low gross domestic") OR (TI "low gross national" OR AB "low gross national") OR (TI "lower gdp" OR AB "lower gdp") OR (TI "lower gnp" OR AB "lower gnp") OR (TI "lower gross domestic" OR AB "lower gross domestic") OR (TI "lower gross national" OR AB "lower gross national") OR (TI lmic OR AB lmic) OR (TI lmics OR AB lmics) OR (TI "third world" OR AB "third world") OR (TI "lami countr*" OR AB "lami countr*") OR (TI "transitional countr*" OR AB "transitional countr*") OR (TI "emerging economies" OR AB "emerging economies") OR (TI "emerging nation#" OR AB "emerging nation#"))
29. 28 AND 29
30. (MH "Rural Health Services") OR (MH "Rural Health") OR (MH "Rural Health Nursing") OR (MH "Rural Health Personnel") OR (MH "Medically Underserved Area") OR (MH "Hospitals, Rural") OR (MH " Rural Health Centers") OR (MH "Rural Population") OR (MH “agriculture”) OR (MH “Farmworkers”) OR (TI rural* OR AB rural*) OR (TI agricultur* OR AB agricultur*) OR (TI rural* OR AB rural*) OR (TI farmer* OR AB farmer*) OR (TI peasant* OR AB peasant*) OR (TI sharecropp* OR AB sharecropp*) OR (((TI remote OR AB remote) OR (TI nonmetropolitan OR AB nonmetropolitan) OR (TI "non metropolitan" OR AB "non metropolitan") OR (TI suburb* OR AB suburb*) OR (TI developing OR AB developing) OR (TI "less* developed" OR AB "less* developed") OR (TI "under developed" OR AB "under developed") OR (TI underdeveloped OR AB underdeveloped) OR (TI underserved OR AB underserved) OR (TI "under served" OR AB "under served") OR (TI deprived OR AB deprived) OR (TI poor OR AB poor)) N3 ((TI communit* OR AB communit*) OR (TI area# OR AB area#) OR (TI village* OR AB village*) OR (TI region# OR AB region#) OR (TI setting* OR AB setting*))) OR (((TI remote OR AB remote) OR (TI nonmetropolitan OR AB nonmetropolitan) OR (TI "non metropolitan" OR AB "non metropolitan") OR (TI underserved OR AB underserved) OR (TI "under served" OR AB "under served") OR (TI deprived OR AB deprived) OR (TI village* OR AB village*)) N3 (TI population* OR AB population*)) OR (((TI remote OR AB remote) OR (TI regional OR AB regional) OR (TI nonmetropolitan OR AB nonmetropolitan) OR (TI "non metropolitan" OR AB "non metropolitan") OR (TI village* OR AB village*)) N3 ((TI clinic# OR AB clinic#) OR (TI hospital# OR AB hospital#) OR (TI facility OR AB facility) OR (TI facilities OR AB facilities) OR (TI "health* center#" OR AB "health* center#") OR (TI "health* centre#" OR AB "health* centre#") OR (TI "health care center#" OR AB "health care center#") OR (TI "health care centre#" OR AB "health care centre#") OR (TI "medical center#" OR AB "medical center#") OR (TI "medical centre#" OR AB "medical centre#")))
31. 30 AND 31
32. (MH "aged+") OR (MH "ageism") OR (MH "attitude to aging") OR (MH "aging+") OR (MH "healthy aging") OR (MH "dental care for older persons") OR (MH "geriatrics+") OR (MH "health services for older persons") OR (MH "gerontologic care") OR (MH "gerontologic nursing+") OR (MH "rehabilitation, geriatric") OR (ag#ing OR elder* OR frail* OR ((aged OR old* OR senior) W1 (adult* OR citizen* OR client* OR group* OR human* OR individual* OR inpatient* OR man OR men OR outpatient* OR patient* OR people OR person* OR population* OR resident* OR wom*n)) OR centenarian* OR "center* for the aged" OR "centre* for the aged" OR geriatric* OR gerontolog* OR "long term care" OR nonagenarian* OR octogenarian* OR "old* age*" OR retir* OR "senior center*" OR "senior centre*" OR seniors OR septuagenarian* OR sexagenarian* OR supercentenarian* OR ((69 OR 70 OR 74 OR 75 OR 79 OR 80 OR 84 OR 85 OR 89 OR 90 OR 94 OR 95) W1 (aged OR year*1)) OR ((60 OR 65) N2 (above OR older OR over OR plus)))
33. 32 AND 33
34. (((TI lived OR AB lived) OR (TI life OR AB life) OR (TI personal* OR AB personal*) OR (TI patient# OR AB patient#) OR (TI survivor* OR AB survivor*)) N3 ((TI experience* OR AB experience*) OR (TI perspective* OR AB perspective*) OR (TI perception* OR AB perception*) OR (TI meaning* OR AB meaning*) OR (TI view# OR AB view#) OR (TI viewpoint* OR AB viewpoint*)))
35. (MH "health behavior+") OR (MH "health services accessibility")
36. (MH "Professional-Patient Relations+") OR (MH "Professional-Client Relations+") OR (MH "Professional-Family Relations")
37. (MH "Diagnosis, Delayed") OR (MH "Health Services Misuse") OR (MH "Treatment Withdrawal") OR (MH "Continuity of Patient Care+") OR (MH "Attitude to Health+") OR (MH "Attitude to Health Personnel+") OR (MH "Attitude to Illness") OR (MH "Office Visits")
38. ((((TI care OR AB care) OR (TI diagnos* OR AB diagnos*) OR (TI healthcare OR AB healthcare) OR (TI health OR AB health) OR (TI treatment* OR AB treatment*)) N2 ((TI access* OR AB access*) OR (TI seek* OR AB seek*))) OR (TI Screening OR AB Screening) OR ((TI disease* OR AB disease*) W1 (TI test* OR AB test*)) OR ((TI Receiv* OR AB Receiv*) W1 ((TI diagnos* OR AB diagnos*) OR (TI therap* OR AB therap*) OR (TI treatment* OR AB treatment*))) OR (((TI Use OR AB Use) OR (TI utili#ation OR AB utili#ation)) N2 ((TI care OR AB care) OR (TI healthcare OR AB healthcare) OR (TI service* OR AB service*))) OR (TI "Patient follow-up" OR AB "Patient follow-up") OR (TI Cope* OR AB Cope*) OR (TI Coping OR AB Coping) OR (((TI adher* OR AB adher*) OR (TI Compliance OR AB Compliance) OR (TI concordan* OR AB concordan*)) W1 ((TI drug* OR AB drug*) OR (TI medication* OR AB medication*))) OR (((TI adher* OR AB adher*) OR (TI Compliance OR AB Compliance) OR (TI concordan* OR AB concordan*)) N3 ((TI therap* OR AB therap*) OR (TI treatment* OR AB treatment*))) OR (TI Underus* OR AB Underus*) OR (TI "under us*" OR AB "under us*"))
39. 35 OR 36 OR 37 OR 38
40. 34 AND 39
41. (TI "cross-sectional study" OR TI "case-control study" OR TI "cohort study" OR TI "multilevel analysis" OR TI cost-effectiveness OR TI cost-utility OR TI "economic evaluation*")
42. (PT guideline OR PT "practice guideline") OR (MH "Practice Guidelines") OR PT review OR (MH " literature review+") OR PT "systematic review" OR PT comment OR PT editorial OR PT meta-analysis OR (MH "meta-analysis") OR TI "systematic review" OR TI "literature review" OR TI "meta analysis" OR TI "scoping review" OR TI "rapid review"
43. 41 OR 42
44. 40 NOT 43
45. Limiters - Published Date: 20020101-20241231

PsycINFO

1. (MH " Qualitative Studies+")
2. ((TI qualitative OR AB qualitative) W1 ((TI research OR AB research) OR (TI stud*3OR AB stud*3)))
3. ((TI mixed OR AB mixed) W1 ((TI method* OR AB method*) OR (TI studies OR AB studies)))
4. (MH " Interviews+")
5. (TI interview* OR AB interview*)
6. (MH "focus groups")
7. (TI "focus group*" OR AB "focus group*")
8. (((TI action OR AB action) OR (TI participatory OR AB participatory)) AND (TI research OR AB research))
9. (MH "Community-Based Participatory Research+")
10. (MH "grounded theory")
11. (TI "grounded theory" OR AB "grounded theory")
12. (TI phenomenolog* OR AB phenomenolog*)
13. (MH "Narratives+")
14. (MH "Observational Methods+")
15. (TI narrat* OR AB narrat*)
16. (TI conversation* OR AB conversation*)
17. (TI discourse* OR AB discourse*)
18. ((TI ethnograph* OR AB ethnograph*) OR (TI ethnomethodolog* OR AB ethnomethodolog*) OR (TI "ethnomethodolog*" OR AB "ethnomethodolog*") OR (TI autoethnograph*OR AB autoethnograph*))
19. (TI hermeneutic* OR AB hermeneutic*)
20. (TI constructivis* OR AB constructivis*)
21. (((TI case OR AB case) OR (TI fi eld OR AB fi eld)) W1 ((TI study OR AB study) OR (TI studies OR AB studies)))
22. (((TI participant* OR AB participant*) OR (TI field OR AB field)) W1 (TI observ* OR AB observ*))
23. (((TI purpos* OR AB purpos*) OR (TI theoretical OR AB theoretical) OR (TI judg#ment OR AB judg#ment) OR (TI "maximum variation" OR AB "maximum variation")OR (TI convenience OR AB convenience) OR (TI "critical case" OR AB "critical case") OR (TI "deviant case" OR AB "deviant case") OR (TI "key informant" OR AB "key informant") OR (TI snowball OR AB snowball) OR (TI cluster OR AB cluster)) W1 (TI sampl* OR AB sampl*))
24. (TI experience* OR TI perspective* OR TI perception* OR TI meaning* OR TI view# OR TI viewpoint*)
25. (((TI lived OR AB lived)OR (TI life OR AB life) OR(TI personal* OR AB personal*) OR (TI patient#OR AB patient#) OR (TI survivor* OR AB survivor*)) N3 ((TI experience* OR AB experience*) OR (TI perspective* OR AB perspective*) OR (TI perception* OR AB perception*) OR (TI meaning* OR AB meaning*) OR (TI view# OR AB view#) OR (TI viewpoint* OR AB viewpoint*)))
26. (((TI thematic OR AB thematic) OR (TI content OR AB content)) W1 (TI analys* OR AB analys*)) (TI "group discussion*"OR AB "group discussion*")
27. S1 OR S2 OR S3 OR S4 OR S5 OR S6 OR S7 OR S8 OR S9 OR S10 OR S11 OR S12 OR S13 OR S14 OR S15 OR S16 OR S17 OR S18 OR S19 OR S20 OR S21 OR S22 OR S23 OR S24 OR S25 OR S26
28. ((TI afghanistan OR AB afghanistan) OR (TI albania OR AB albania)OR (TI algeria OR AB algeria) OR (TI "americansamoa" OR AB "americansamoa") OR (TI angola OR AB angola) OR (TI"antigua and barbuda" OR AB "antigua andbarbuda") OR (TI antigua OR AB antigua) OR (TI barbuda OR AB barbuda)OR (TI argentina OR AB argentina) OR (TI armenia OR AB armenia) OR (TI armenian OR AB armenian) OR (TI aruba OR AB aruba) OR (TI azerbaijan OR AB azerbaijan) OR (TI bahrain OR AB bahrain)OR (TI bangladesh OR AB bangladesh) OR (TI barbados OR AB barbados) OR (TI "republic of belarus" OR AB "republic of belarus")OR (TI belarus OR AB belarus) OR (TI byelarus OR AB byelarus) OR (TI belorussia OR AB belorussia) OR (TI byelorussian OR AB byelorussian) OR (TI belize OR AB belize) OR(TI "british honduras" OR AB "british honduras") OR(TI benin OR AB benin)OR (TI dahomey OR AB dahomey) OR (TI bhutan OR AB bhutan) OR (TI bolivia OR AB bolivia) OR(TI "bosnia andherzegovina" OR AB "bosnia and herzegovina")OR (TI bosnia OR AB bosnia) OR (TI herzegovina OR AB herzegovina) OR (TI botswana OR AB botswana) OR (TI bechuanaland OR AB bechuanaland) OR (TI brazil OR AB brazil) OR(TI brasil OR AB brasil)OR (TI bulgaria OR AB bulgaria) OR (TI "burkinafaso" OR AB "burkinafaso") OR (TI "burkinafasso" OR AB "burkinafasso") OR (TI "uppervolta" OR AB "uppervolta") OR (TI burundi OR AB burundi) OR (TI urundi OR AB urundi) OR (TI"cabo verde" OR AB"cabo verde") OR (TI "cape verde" OR AB "cape verde") OR (TI cambodia OR AB cambodia) OR (TI kampuchea OR AB kampuchea) OR (TI "khmer republic" OR AB"khmer republic") OR (TI cameroon OR AB cameroon) OR (TI cameron OR AB cameron) OR (TI cameroun OR AB cameroun) OR (TI "centralafrican republic" OR AB "central african republic")OR (TI "ubangi shari" OR AB "ubangi shari") OR (TI chad OR AB chad) OR (TI chile OR AB chile) OR (TI china OR AB china) OR(TI colombia OR AB colombia) OR (TI comoros OR AB comoros) OR (TI"comoro islands" OR AB"comoro islands") OR (TI"iles comers" OR AB "ilescomers") OR (TI mayotte OR AB mayotte) OR (TI "democratic republic of the congo" OR AB "democratic republic of the congo") OR (TI "democratic republic congo" OR AB "democratic republic congo") OR (TI congo OR AB congo) OR (TI zaire OR AB zaire) OR (TI"costa rica" OR AB "costarica") OR (TI "coted’ivoire" OR AB "coted’ivoire") OR (TI "cote d’ivoire" OR AB "cote d’ivoire") OR (TI "cotedivoire" OR AB "cotedivoire") OR (TI "cote divoire" OR AB "cote divoire") OR (TI "ivorycoast" OR AB "ivorycoast") OR (TI croatia OR AB croatia) OR (TI cuba OR AB cuba) OR (TI cyprus OR AB cyprus) OR(TI "czech republic" OR AB "czech republic") OR(TI czechoslovakia OR AB czechoslovakia) OR (TI djibouti OR AB djibouti)OR (TI "frenchsomaliland" OR AB"french somaliland") OR(TI dominica OR AB dominica) OR (TI"dominican republic" OR AB "dominican republic")OR (TI ecuador OR AB ecuador) OR (TI egypt OR AB egypt) OR (TI "unitedarab republic" OR AB"united arab republic") OR(TI "el salvador" OR AB"el salvador") OR (TI"equatorial guinea" OR AB "equatorial guinea")OR (TI "spanish guinea"OR AB "spanish guinea")OR (TI eritrea OR AB eritrea) OR (TI estonia OR AB estonia) OR (TI eswatini OR AB eswatini)OR (TI swaziland OR AB swaziland) OR (TI ethiopia OR AB ethiopia)OR (TI fi ji OR AB fi ji) OR(TI gabon OR AB gabon)OR (TI "gaboneserepublic" OR AB"gabonese republic") OR(TI gambia OR AB gambia) OR (TI "georgia(republic)" OR AB"georgia (republic)") OR(TI georgian OR AB georgian) OR (TI ghana OR AB ghana) OR (TI"gold coast" OR AB "goldcoast") OR (TI gibraltarOR AB gibraltar) OR (TI greece OR AB greece)OR (TI grenada OR AB grenada) OR (TI guam OR AB guam) OR (TI guatemala OR AB guatemala) OR (TI guinea OR AB guinea) OR (TI"guinea bissau" OR AB"guinea bissau") OR (TI guyana OR AB guyana)OR (TI "british guiana" OR AB "british guiana") OR(TI haiti OR AB haiti) OR(TI hispaniola OR AB hispaniola) OR (TI honduras OR AB honduras) OR (TI hungaryOR AB hungary) OR (TI india OR AB india) OR (TI indonesia OR AB indonesia) OR (TI timorOR AB timor) OR (TI iran OR AB iran) OR (TI iraqOR AB iraq) OR (TI "isleof man" OR AB "isle ofman") OR (TI jamaica OR AB jamaica) OR (TI jordan OR AB jordan) OR (TI kazakhstan OR ABkazakhstan) OR (TI kazakh OR AB kazakh)OR (TI kenya OR ABkenya) OR (TI"democratic people’srepublic of korea" OR AB"democratic people’srepublic of korea") OR (TI"north korea" OR AB"north korea") OR (TI kosovo OR AB kosovo)OR (TI kyrgyzstan OR AB kyrgyzstan) OR (TI kirghizia OR AB kirghizia)OR (TI kirgizstan OR AB kirgizstan) OR (TI "kyrgyzrepublic" OR AB "kyrgyzrepublic") OR (TI kirghizOR AB kirghiz) OR (TI laos OR AB laos) OR (TI"lao pdr" OR AB "lao pdr")OR (TI "lao people's democratic republic" OR AB "lao people's democratic republic") OR(TI latvia OR AB latvia)OR (TI lebanon OR AB lebanon) OR (TI"lebanese republic" OR AB "lebanese republic")OR (TI lesotho OR ABlesotho) OR (TI basutoland OR AB basutoland) OR (TI liberia OR AB liberia) OR (TI libya OR AB libya) OR (TI"libyan arab jamahiriya"OR AB "libyan arabjamahiriya") OR (TI lithuania OR AB lithuania)OR (TI macau OR AB macau) OR (TI macao OR AB macao) OR (TI"republic of northmacedonia" OR AB"republic of northmacedonia") OR (TI macedonia OR AB macedonia) OR (TI madagascar OR AB madagascar) OR (TI"malagasy republic" OR AB "malagasy republic")OR (TI malawi OR AB malawi) OR (TI nyasalandOR AB nyasaland) OR (TI malaysia OR AB malaysia) OR (TI "malayfederation" OR AB "malay federation") OR (TI"malaya federation" OR AB "malaya federation")OR (TI maldives OR AB maldives) OR (TI "indianocean islands" OR AB"indian ocean islands")OR (TI "indian ocean" OR AB "indian ocean") OR (TI mali OR AB mali) OR (TI malta OR AB malta) OR(TI micronesia OR AB micronesia) OR (TI"federated states ofmicronesia" OR AB"federated states ofmicronesia") OR (TI kiribati OR AB kiribati) OR(TI "marshall islands" OR AB "marshall islands") OR(TI nauru OR AB nauru)OR (TI "northern marianaislands" OR AB "northernmariana islands") OR (TI palau OR AB palau) OR(TI tuvalu OR AB tuvalu)OR (TI mauritania OR AB mauritania) OR (TI mauritius OR AB mauritius) OR (TI mexicoOR AB mexico) OR (TI moldova OR AB moldova)OR (TI moldovian OR AB moldovian) OR (TI mongolia OR AB mongolia) OR (TI montenegro OR AB montenegro) OR (TI morocco OR AB morocco)OR (TI ifni OR AB ifni) OR(TI mozambique OR AB mozambique) OR (TI"portuguese east africa"OR AB "portuguese east africa") OR (TI myanmarOR AB myanmar) OR (TI burma OR AB burma) OR(TI namibia OR ABnamibia) OR (TI nepal OR AB nepal) OR (TI"netherlands antilles" OR AB "netherlands antilles")OR (TI nicaragua OR ABnicaragua) OR (TI nigerOR AB niger) OR (TI nigeria OR AB nigeria)OR (TI oman OR ABoman) OR (TI muscat OR AB muscat) OR (TI pakistan OR AB pakistan)OR (TI panama OR ABpanama) OR (TI "papuanew guinea" OR AB"papua new guinea") OR(TI "new guinea" OR AB"new guinea") OR (TI paraguay OR ABparaguay) OR (TI peruOR AB peru) OR (TI philippines OR ABphilippines) OR (TI philipines OR ABphilipines) OR (TI phillipines OR ABphillipines) OR (TI phillippines OR ABphillippines) OR (TI poland OR AB poland)OR (TI "polish people'srepublic" OR AB "polishpeople's republic") OR (TI"puerto rico" OR AB"puerto rico") OR (TI romania OR AB romania)OR (TI russia OR ABrussia) OR (TI "russianfederation" OR AB"russian federation") OR (TI ussr OR AB ussr) OR(TI "soviet union" OR AB"soviet union") OR (TI"union of soviet socialistrepublics" OR AB "unionof soviet socialistrepublics") OR (TI rwanda OR AB rwanda) OR (TI ruanda OR AB ruanda)OR (TI samoa OR AB samoa) OR (TI "pacifi cislands" OR AB "pacifi cislands") OR (TI polynesia OR AB polynesia) OR (TI"samoan islands" OR AB"samoan islands") OR (TI"navigator island" OR AB"navigator island") OR (TI"navigator islands" OR AB"navigator islands") OR(TI "sao tome andprincipe" OR AB "saotome and principe") OR(TI "saudi arabia" OR AB"saudi arabia") OR (TI senegal OR AB senegal)OR (TI serbia OR AB serbia) OR (TI seychelles OR AB seychelles) OR (TI"sierra leone" OR AB"sierra leone") OR (TI slovakia OR AB slovakia)OR (TI "slovak republic"OR AB "slovak republic")OR (TI slovenia OR AB slovenia) OR (TI melanesia OR AB melanesia) OR (TI"solomon island" OR AB"solomon island") OR (TI"solomon islands" OR AB"solomon islands") OR (TI"norfolk island" OR AB"norfolk island") OR (TI "norfolk islands" OR AB"norfolk islands") OR (TI somalia OR AB somalia)OR (TI "south africa" OR AB "south africa") OR (TI"south sudan" OR AB"south sudan") OR (TI "srilanka" OR AB "sri lanka")OR (TI ceylon OR AB ceylon) OR (TI "saint kittsand nevis" OR AB "saintkitts and nevis") OR (TI"st. kitts and nevis" OR AB "st. kitts and nevis")OR (TI "saint lucia" OR AB "saint lucia") OR (TI"st. lucia" OR AB "st.lucia") OR (TI "saintvincent and thegrenadines" OR AB "saintvincent and thegrenadines") OR (TI "saintvincent" OR AB "saintvincent") OR (TI "st.vincent" OR AB "st.vincent") OR (TI grenadines OR AB grenadines) OR (TI sudan OR AB sudan) OR (TI suriname OR AB suriname) OR (TI surinam OR AB surinam) OR (TI"dutch guiana" OR AB"dutch guiana") OR (TI"netherlands guiana" OR AB "netherlands guiana")OR (TI syria OR AB syria)OR (TI "syrian arabrepublic" OR AB "syrianarab republic") OR (TI tajikistan OR ABtajikistan) OR (TI tadjikistan OR ABtadjikistan) OR (TI tadzhikistan OR ABtadzhikistan) OR (TI tadzhik OR AB tadzhik)OR (TI tanzania OR ABtanzania) OR (TI tanganyika OR ABtanganyika) OR (TI thailand OR AB thailand)OR (TI siam OR AB siam)OR (TI "timor leste" OR AB "timor leste") OR (TI"east timor" OR AB "easttimor") OR (TI togo OR AB togo) OR (TI "togolese republic" OR AB "togolese republic") OR (TI tonga OR AB tonga) OR (TI "trinidad and tobago" OR AB "trinidad and tobago")OR (TI trinidad OR ABtrinidad) OR (TI tobagoOR AB tobago) OR (TI tunisia OR AB tunisia) OR(TI turkey OR AB turkey)OR (TI turkmenistan OR AB turkmenistan) OR (TI turkmen OR AB turkmen)OR (TI uganda OR ABuganda) OR (TI ukraine OR AB ukraine) OR (TI uruguay OR AB uruguay)OR (TI uzbekistan OR ABuzbekistan) OR (TI uzbekOR AB uzbek) OR (TI vanuatu OR AB vanuatu)OR (TI "new hebrides" OR AB "new hebrides") OR(TI venezuela OR ABvenezuela) OR (TI vietnam OR AB vietnam)OR (TI "viet nam" OR AB"viet nam") OR (TI "middleeast" OR AB "middleeast") OR (TI "west bank" OR AB "west bank") OR(TI gaza OR AB gaza) OR(TI palestine OR ABpalestine) OR (TI yemen OR AB yemen) OR (TI yugoslavia OR AByugoslavia) OR (TI zambia OR AB zambia)OR (TI zimbabwe OR ABzimbabwe) OR (TI"northern rhodesia" OR AB "northern rhodesia")OR (TI "global south" OR AB "global south") OR (TI"africa south of thesahara" OR AB "africasouth of the sahara") OR(TI "sub-saharan africa"OR AB "sub-saharanafrica") OR (TI"subsaharan africa" OR AB "subsaharan africa")OR (TI "africa, central"OR AB "africa, central")OR (TI "central africa" OR AB "central africa") OR (TI"africa, northern" OR AB"africa, northern") OR (TI"north africa" OR AB"north africa") OR (TI"northern africa" OR AB"northern africa") OR (TI magreb OR AB magreb)OR (TI maghrib OR AB maghrib) OR (TI sahara OR AB sahara) OR (TI"africa, southern" OR AB"africa, southern") OR (TI"southern africa" OR AB"southern africa") OR (TI"africa, eastern" OR AB"africa, eastern") OR (TI"east africa" OR AB "eastafrica") OR (TI "eastern africa" OR AB "easternafrica") OR (TI "africa,western" OR AB "africa,western") OR (TI "westafrica" OR AB "westafrica") OR (TI "westernafrica" OR AB "westernafrica") OR (TI "westindies" OR AB "westindies") OR (TI "indianocean islands" OR AB"indian ocean islands")OR (TI caribbean OR AB caribbean) OR (TI "centralamerica" OR AB "centralamerica") OR (TI "latinamerica" OR AB "latinamerica") OR (TI "southand central america" OR AB "south and centralamerica") OR (TI "southamerica" OR AB "southamerica") OR (TI "asia,central" OR AB "asia,central") OR (TI "centralasia" OR AB "centralasia") OR (TI "asia,northern" OR AB "asia,northern") OR (TI "northasia" OR AB "north asia")OR (TI "northern asia" OR AB "northern asia") OR(TI "asia, southeastern"OR AB "asia,southeastern") OR (TI"southeastern asia" OR AB "southeastern asia")OR (TI "south easternasia" OR AB "southeastern asia") OR (TI"southeast asia" OR AB"southeast asia") OR (TI"south east asia" OR AB"south east asia") OR (TI "asia, western" OR AB"asia, western") OR (TI"western asia" OR AB"western asia") OR (TI"europe, eastern" OR AB"europe, eastern") OR (TI"east europe" OR AB"east europe") OR (TI"eastern europe" OR AB"eastern europe") OR (TI"developing country" OR AB "developing country")OR (TI "developingcountries" OR AB"developing countries")OR (TI "developingnation#" OR AB"developing nation#") OR(TI "developingpopulation#" OR AB"developing population#")OR (TI "developing world "OR AB "developing world") OR (TI "less developed countr*" OR AB "less developed countr*") OR (TI "lessdeveloped nation#" OR AB "less developednation#") OR (TI "lessdeveloped population#"OR AB "less developedpopulation#") OR (TI "lessdeveloped world" OR AB"less developed world")OR (TI "lesser developedcountr*" OR AB "lesserdeveloped countr*") OR(TI "lesser developednation#" OR AB "lesserdeveloped nation#") OR(TI "lesser developedpopulation#" OR AB"lesser developed population#") OR (TI"lesser developed world"OR AB "lesser developedworld") OR (TI "underdeveloped countr*" OR AB "under developedcountr*") OR (TI "underdeveloped nation#" OR AB "under developednation#") OR (TI "underdeveloped population#"OR AB "under developedpopulation#") OR (TI"under developed world"OR AB "under developedworld") OR (TI"underdeveloped countr*"OR AB "underdevelopedcountr*") OR (TI"underdeveloped nation#"OR AB "underdevelopednation#") OR (TI"underdevelopedpopulation#" OR AB"underdevelopedpopulation#") OR (TI"underdeveloped world"OR AB "underdevelopedworld") OR (TI "middleincome countr*" OR AB"middle income countr*")OR (TI "middle incomenation#" OR AB "middleincome nation#") OR (TI"middle incomepopulation#" OR AB"middle incomepopulation#") OR (TI "lowincome countr*" OR AB"low income countr*") OR(TI "low income nation#"OR AB "low incomenation#") OR (TI "lowincome population#" OR AB "low incomepopulation#") OR (TI"lower income countr*"OR AB "lower incomecountr*") OR (TI "lowerincome nation#" OR AB"lower income nation#")OR (TI "lower incomepopulation#" OR AB"lower incomepopulation#") OR (TI"underserved countr*" OR AB "underserved countr*")OR (TI "underservednation#" OR AB"underserved nation#")OR (TI "underservedpopulation#" OR AB"underservedpopulation#") OR (TI"underserved world" OR AB "underserved world")OR (TI "under servedcountr*" OR AB "underserved countr*") OR (TI"under served nation#"OR AB "under servednation#") OR (TI "underserved population#" OR AB "under servedpopulation#") OR (TI"under served world" OR AB "under served world")OR (TI "deprived countr*"OR AB "deprived countr*")OR (TI "deprived nation#"OR AB "deprivednation#") OR (TI "deprivedpopulation#" OR AB"deprived population#")OR (TI "deprived world"OR AB "deprived world")OR (TI "poor countr*" OR AB "poor countr*") OR (TI "poor nation#" OR AB"poor nation#") OR (TI"poor population#" OR AB"poor population#") OR(TI "poor world" OR AB"poor world") OR (TI"poorer countr*" OR AB"poorer countr*") OR (TI"poorer nation#" OR AB"poorer nation#") OR (TI"poorer population#" OR AB "poorer population#")OR (TI "poorer world" OR AB "poorer world") OR (TI"developing econom*" OR AB "developing econom*")OR (TI "less developedeconom*" OR AB "lessdeveloped econom*") OR(TI "lesser developedeconom*" OR AB "lesserdeveloped econom*") OR(TI "under developedeconom*" OR AB "underdeveloped econom*") OR(TI "underdevelopedeconom*" OR AB"underdevelopedeconom*") OR (TI "middleincome econom*" OR AB"middle income econom*")OR (TI "low incomeeconom*" OR AB "lowincome econom*") OR (TI"lower income econom*"OR AB "lower incomeeconom*") OR (TI "lowgdp" OR AB "low gdp")OR (TI "low gnp" OR AB"low gnp") OR (TI "lowgross domestic" OR AB"low gross domestic") OR(TI "low gross national"OR AB "low gross national") OR (TI "lowergdp" OR AB "lower gdp")OR (TI "lower gnp" OR AB"lower gnp") OR (TI "lowergross domestic" OR AB"lower gross domestic")OR (TI "lower grossnational" OR AB "lowergross national") OR (TI lmic OR AB lmic) OR (TI lmics OR AB lmics) OR(TI "third world" OR AB"third world") OR (TI "lamicountr*" OR AB "lamicountr*") OR (TI"transitional countr*" OR AB "transitional countr*")OR (TI "emergingeconomies" OR AB"emerging economies")OR (TI "emerging nation#"OR AB "emergingnation#"))
29. S27 AND S28
30. (MH "Rural Health Services") OR (MH "Rural Health") OR (MH "Rural Health Nursing") OR (MH "Rural Health Personnel")OR (MH "Suburban Health") OR (MH "Medically Underserved Area") OR (MH "Hospitals, Rural") OR(MH " Rural Health Centers")OR (MH "Rural Population") OR (MH "Suburban Population") OR rural* OR (((TI remote OR AB remote) OR (TI nonmetropolitan OR AB nonmetropolitan) OR (TI "non metropolitan" OR AB "non metropolitan") OR(TI suburb* OR AB suburb*) OR (TI developing OR AB developing) OR (TI "less*developed" OR AB "less*developed") OR (TI "under developed" OR AB "under developed") OR(TI underdeveloped ORAB underdeveloped) OR(TI "middle income" ORAB "middle income") OR(TI "low* income" OR AB "low* income") OR (TI underserved OR AB underserved) OR (TI"under served" OR AB"under served") OR (TI deprived OR AB deprived)OR (TI poor OR AB poor))N3 ((TI communit* OR AB communit*) OR (TI area# OR AB area#) OR (TI village* OR AB village*)OR (TI region# OR AB region#) OR (TI province# OR AB province#) OR (TI setting* OR AB setting*)))OR (((TI remote OR AB remote) OR (TI nonmetropolitan OR AB nonmetropolitan) OR (TI "non metropolitan" OR AB "non metropolitan") OR(TI suburb* OR AB suburb*) OR (TI "middle income" OR AB "middle income") OR (TI "low*income" OR AB "low*income") OR (TI underserved OR AB underserved) OR (TI "under served" OR AB" under served") OR (TI deprived OR AB deprived)OR (TI poor OR AB poor)OR (TI village* OR AB village*)) N3 (TI population* OR AB population*)) OR (((TI remote OR AB remote)OR (TI regional OR AB regional) OR (TI nonmetropolitan OR AB nonmetropolitan) OR (TI "non metropolitan" OR AB "non metropolitan") OR(TI suburb* OR AB suburb*) OR (TI village*OR AB village*)) N3 ((TI clinic# OR AB clinic#) OR(TI hospital# OR AB hospital#) OR (TI facility OR AB facility) OR (TI facilities OR AB facilities)OR (TI "health* center#"OR AB "health* center#")OR (TI "health* centre#" OR AB "health* centre#")OR (TI "health care center#" OR AB "healthcare center#") OR (TI "health care centre#" ORAB "health care centre#")OR (TI "medical center#"OR AB "medical center#")OR (TI "medical centre#" OR AB "medical centre#"))) OR (((TI shortage OR AB shortage) OR (TI understaffed OR AB understaffed) OR (TI"under staffed" OR AB "under staffed")) N3 (TI area# OR AB area#))
31. S29 AND S30
32. (MH "aged+") OR (MH "ageism") OR (MH "attitude to aging") OR(MH "aging+") OR (MH "healthy aging") OR (MH "dental care for older persons") OR (MH "geriatrics+") OR (MH"health services for older persons") OR (MH"gerontologic care") OR(MH "gerontologic nursing+") OR (MH "rehabilitation, geriatric")OR (ag#ing OR elder* OR frail* OR ((aged OR "high risk" OR old* OR senior OR vulnerable) W1 (adult*OR citizen* OR client* OR group* OR human* OR individual* OR inpatient*OR man OR men OR outpatient* OR patient*OR people OR person*OR population* OR resident* OR wom*n)) OR centenarian* OR "center* for the aged" OR "centre*for the aged" OR geriatric*OR gerontolog* OR "long term care" OR nonagenarian* OR octogenarian* OR "old*age*" OR retir* OR "seniorcenter*" OR "seniorcentre*" OR seniors OR septuagenarian* OR sexagenarian* OR supercentenarian* OR veteran* OR ((69 OR 70OR 74 OR 75 OR 79 OR80 OR 84 OR 85 OR 89OR 90 OR 94 OR 95) W1(aged OR year*1)) OR((60 OR 65) N2 (above OR older OR over OR plus)))
33. S31 AND S32
34. (MH "health behavior+") OR (MH "health services accessibility")
35. (MH "Professional-Patient Relations+") OR (MH "Professional-Client Relations+") OR (MH "Professional-Family Relations")
36. (MH "Diagnosis, Delayed") OR (MH "Health Services Misuse") OR (MH "Treatment Withdrawal") OR (MH "Continuity of Patient Care+") OR (MH "Attitude to Health+") OR (MH "Attitude to Health Personnel+") OR (MH "Attitude to Illness") OR(MH "Office Visits")
37. ((((TI care OR AB care)OR (TI diagnos* OR AB diagnos*) OR (TI healthcare OR AB healthcare) OR (TI health OR AB health) OR (TI treatment* OR AB treatment*)) N2 ((TI access* OR AB access*)OR (TI seek* OR AB seek*))) OR (TI Screening OR AB Screening) OR((TI disease* OR AB disease*) W1 (TI test* ORAB test*)) OR ((TI Receiv*OR AB Receiv*) W1 ((TI diagnos* OR AB diagnos*) OR (TI therap*OR AB therap*) OR (TI treatment* OR AB treatment*))) OR (((TI Use OR AB Use) OR (TI utili#ation OR AB utili#ation)) N2 ((TI care OR AB care) OR (TI healthcare OR AB healthcare) OR (TI service* OR AB service*))) OR (TI "Patient follow-up" OR AB "Patient follow-up") OR (TI Cope*OR AB Cope*) OR (TI Coping OR AB Coping)OR (TI Compliance OR AB Compliance) OR (TI concordan* OR AB concordan*) OR (((TI adher* OR AB adher*) OR(TI take OR AB take) OR(TI taking OR AB taking))W1 ((TI drug* OR AB drug*) OR (TI medication*OR AB medication*))) OR((TI adher* OR AB adher*)N3 ((TI therap* OR AB therap*) OR (TI treatment*OR AB treatment*))) OR(TI Underus* OR AB Underus*) OR (TI "underus*" OR AB "under us*"))
38. S34 OR S35 OR S36 OR S37
39. S33 AND S38
40. (TI "cross-sectional study" OR TI "case-control study" OR TI "cohort study" OR TI "multilevel analysis" OR TI cost-effectiveness OR TI cost-utility OR TI "economic evaluation*")
41. (PT guideline OR PT "practice guideline") OR (MH "Practice Guidelines") OR PT review OR (MH " literature review+") OR PT "systematic review" ORPT comment OR PT editorial OR PT meta-analysis OR (MH "meta-analysis") OR TI "systematic review" OR TI "literature review" OR TI "meta analysis" OR TI "scoping review" OR TI "rapid review"
42. S41 or S40
43. S39 not S42
44. Limiters- Published Date:20020101-20241231. Expanders - Apply related words; Apply equivalent subjects

# **Supplemental** **Appendix 2: Hierarchical screening protocol and form used for review of full-text studies**

1. Does the study take place in the low- and middle-income countries (LMICs)? (Yes/No)

2. Are the participants elderly patients? (Yes/No)

a) If participant sample is mixed (young people + elderly people), exclude unless the focus of the study is on the differences among different age groups or the study has a clear description on the health-seeking patterns of the elderly participants

3. Do the study participants live in a rural area? (Yes/No)

a) Note: "tribal" almost always refer to rural area unless otherwise stated

b) If participant sample is mixed (rural + urban + suburban), exclude unless the focus of the study is on rural-urban differences

4. Is the study qualitative (prioritizes descriptive findings on patient experiences, perceptions, or attitudes)? (Yes/No)

a) Exclude purely quantitative studies, systematic reviews, and non-empirical articles (e.g., editorials)

5. Does that study focus on health-seeking to or use of health services? (Yes/No)

a) “Health-seeking behaviours” = A process of how patients engage with health system within certain social systems, cultural norms and system constraints

b) "Health services" = Health care and clinical services or other community-based health services provided by a public or private organization

c) The study's research question/focus should reflect the focus of the review: How rural elderly patients experience and understand their experience of health-care seeking in LMICs? Based on these patients’ perspective, what are the barriers and facilitators of their health-care seeking?

d) Exclude studies focused only on participant self-management

6. Are the study participants the patients themselves and/or their caregivers? (Yes/No)

a) Exclude studies where the participants are only clinicians or other health care stakeholders, not the patients or caregivers themselves

# **Supplemental Appendix 3: Data extraction protocol and form**

| General information | Study title |
| --- | --- |
|  | First author (Last name) |
|  | Journal |
|  | Year published |
|  | Year study conducted |
|  | Location (country and province) |
| Participants | Type (Patients, caregivers, or both) |
|  | Chronic disease(s)/health conditions under study |
|  | Number of participants |
|  | Age distribution |
|  | Sex and/or gender distribution |
|  | Race/Ethnicity distribution |
|  | Marital status |
|  | Education |
|  | Insurance status |
|  | Other socioeconomic measurement (e.g., income, employment, etc.) |
| Methodology | Study aims, purpose, or objective |
|  | Theoretical framework (leave blank if unspecified) |
|  | Study design (e.g., qualitative study – narrative, ethnography, grounded theory, case study, phenomenology, etc. mixed method study – exploratory, explanatory, convergent, etc. Leave blank if unspecified) |
|  | Sampling procedure |
|  | Data collection method(s) |
|  | Data collection mode (e.g., in-person, telephone, online. Leave blank if unspecified) |
|  | Data analysis approach (e.g., content analysis, narrative analysis, discourse analysis, grounded theory, etc. Leave blank if unspecified) |

# **Supplemental Appendix 4. Detailed characteristics of 28 eligible articles**

| No | **General information** | | | | | | **Participants** | | | | | | | | | | **Methodology** | | | | | | |
| --- | --- | --- | --- | --- | --- | --- | --- | --- | --- | --- | --- | --- | --- | --- | --- | --- | --- | --- | --- | --- | --- | --- | --- |
|  | Study title | First author (Last name) | Journal | Year published | Year study conducted | Location (country and province) | Type (Patients, caregivers, or both) | Chronic disease(s)/health conditions under study | Number of participants | Age distribution | Sex and/or gender distribution | Race/Ethnicity distribution | Marital status | Education | Insurance status | Other socioeconomic measurement | Study aims, purpose, or objective | Theoretical framework | Study design | Sampling procedure | Data collection method(s) | Data collection mode | Data analysis approach |
| 1 | "When someone becomes old then every part of the body too becomes old": Experiences of living with dementia in Kintampo, rural Ghana ^1^ | Agyeman-Duah | Transcultural Psychiatry | 2019 | 2015 | Kintampo, Ghana | both | dementia | 28 in all (10 patients and some of their co-residents) | mean 84.9; range 73~100 | patients: 6 females (60%), caregivers: 15 females (100%) | NA | NA | NA | patients: National Health Insurance Scheme | NA | To explore the sociocultural beliefs, understandings, perceptions and behaviours relating to living with dementia in Kintampo, Ghana. | NA | case study | Multistage sampling based on screening | in-depth interview | in person | Framework approach |
| 2 | "Let's talk about money": how do poor older people finance their healthcare in rural Ghana? A qualitative study ^2^ | Agyemang-Duah | International Journal for Equity in Health | 2019 | 2018 | Atwima Nwabiagya District, Ghana | both | NA | patients: 30, caregivers: 15 | patients: 65–69: 9; 70–74: 6; 75–79: 3; 80–84: 4; 85–89: 3; 90 or over: 5 | patients: 23 females (77%), caregivers: 15 females (100%) | patients: 25 Akan ethnicity(83.3%), 5 Northerner（16.7%）; caregivers: 13 Akan ethnicity(86.7%), 2 Northerner（13.3%） | NA | 19 had no education | patients: the National Health Insurance Scheme (NHIS) | patients: Christians (27, 90%), living in poverty | Exploring how poor older people finance their healthcare in rural Ghana. | Interpretivist paradigm | Convergent mixed method study design | Purposive and convenience sampling | In-depth interviews and focus group discussions (FGDs) | in person | thematic analysis (posteriori inductive) |
| 3 | Barriers to formal healthcare utilisation among poor older people under the livelihood empowerment against poverty programme in the Atwima Nwabiagya District of Ghana ^3^ | Agyemang-Duah | BMC Public Health | 2019 | NA | Atwima Nwabiagya District, Ghana | both | NA | patients: 30, caregivers: 15 | patients: ≥65 | patients: 23 females (77%) , caregivers: 15 females(100%) | patients: 25 Akan ethnicity(83.3%), 5 Northerner（16.7%）; caregivers: 13 Akan ethnicity(86.7%), 2 Northerner（13.3%） | NA | 19 (63.3%) no level of education, 8 (26.7%) basic, 3 (10%) secondary education | patients: NHIS (National Health Insurance Scheme) | religion: Christianity: 27 (90%) Islam: 3 (10%) Enrolled in the Livelihood Empowerment Against Poverty (LEAP) programme | To explores barriers to formal healthcare use among poor older people under the Livelihood Empowerment Against Poverty (LEAP) programme in the Atwima Nwabiagya District of Ghana. | NA | Narrative research | Purposive and convenience sampling strategies | in-depth interviews and focus group discussions | in person | Thematic analysis (a posteriori inductive method) |
| 4 | Factors influencing the use of public and private health care facilities among poor older people in rural Ghana ^4^ | Agyemang-Duah | Journal of Public Health | 2020 | 2018 | Atwima Nwabiagya District, Ghana | both | NA | patients: 30, caregivers: 15 | patients: ≥65 | patients: 23 females (77%) , caregivers: 15 females(100%) | patients: 25 Akan ethnicity(83.3%), 5 Northerner（16.7%）; caregivers: 13 Akan ethnicity(86.7%), 2 Northerner（13.3%） | NA | 19 (63.3%) no level of education, 8 (26.7%) basic, 3 (10%) secondary education | patients: NHIS (National Health Insurance Scheme) | religion: Christianity: 27 (90%) Islam: 3 (10%) Enrolled in the Livelihood Empowerment Against Poverty (LEAP) programme | To explore issues influencing public and private health care facilities use among poor older people in rural Ghana. | Interpretivist paradigm | Narrative research | Purposive and convenience sampling strategies | In-depth interviews and focus group discussions | in person | A thematic analytical framework |
| 5 | The World Is Not Mine - Barriers to Healthcare Access for Bangladeshi Rural Elderly Women ^5^ | Hamiduzzaman | Journal of cross-cultural gerontology | 2021 | 2015 | Sylhet district, Bangladeshi | patients | 88% (22) experienced at least two diseases and 68% (17) reported at least three, overs the year of data collection | 25 | mean 72; range 60~100 | 25 female (100%) | NA | 20 widowed (80%) | 72% had no formation education | NA | 17 women (68%) having no income; 15 (60%) women were living with extended family | To explore how this cohort related their healthcare access to their living circumstances and provided insight into how their healthcare access needs can be addressed. | A blended critical social model based on Habermas' Theory of Communicative Action and Honneth's Theory of Recognition and Misrecognition | Narrative research | comprehensive sampling | semi-structured interview | in person | critical thematic discourse analysis |
| 6 | When I suffer from fever, I eat mangos. Determinants of health-seeking beliefs and behaviors of rural older women in Sylhet, Bangladesh ^6^ | Hamiduzzaman | Journal of women & aging | 2021 | NA | Sylhet district, Bangladeshi | patients | NA | 25 | 60~100 | 25 female (100%) | NA | 20 widowed (80%), 1 unmarried (4%) , 4 in marital relationships(16%) | 20 (80%) women had no formal education, 5 (20%) had attended school [highest education completed: grade eight] | NA | 18 (72%) women were housewife. 18 (72%) women having no income at all. 15 (60%) women were living with their extended family. | To report the socio-ecological determinants of health-seeking beliefs and behaviors of rural older women in North-eastern Bangladesh and how these behaviors impact their recognition within the setting. | Socio-ecological model | Narrative research | comprehensive sampling | semi-structured interview | in person | thematic analysis |
| 7 | Exploring the System Determinants Associated with Senior Women's Access to Medical Care in Rural Bangladesh ^7^ | Hamiduzzaman | Ageing International | 2021 | NA | Sylhet district, Bangladeshi | patients | NA | 25 | >60 | 25 female (100%) | NA | NA | NA | NA | NA | To explore the system determinants that impact on rural senior women’s utilization of healthcare. | A blended critical social model based on Habermas' Theory of Communicative Action and Honneth's Theory of Recognition and Misrecognition | Narrative research | comprehensive sampling | semi-structured interview | in person | critical thematic discourse analysis |
| 8 | Self-reported Seasonal Symptoms and Diseases and Primary Healthcare Utilization Among Rural Elderly Women in Sylhet District, Bangladesh ^8^ | Hamiduzzaman | Journal of UOEH | 2020 | 2015 | Sylhet district, Bangladeshi | patients | Self-reported Seasonal Symptoms | 65 | mean 72 (SD: 10.2) | 65 female (100%) | NA | NA | 55 (84.6%) No formal education | NA | 48 (73.85) No income | To identify the health effects of seasonal variations that place increased risk of symptoms and diseases on rural elderly women, and to explore the determinants associated with the womenʼs use of healthcare locally. | NA | mixed-methods study | NA | semi-structured interview | in person | thematic analysis |
| 9 | Aging, care and dependency in multimorbidity: how do relationships affect older Bangladeshi women's use of homecare and health services? ^9^ | Hamiduzzaman | Journal of women & aging | 2021 | NA | Sylhet district, Bangladeshi | patients | multimorbidity | 22 | mean 72 (SD 10.21) | 22 female (100%) | NA | 18 widowed (81.8%), 1 unmarried(4.5%) , 3 marital(13.6%) | 15 (68.2%) No schooling, 3 (13.6%) Primary schooling, 4 (18.2%) Secondary schooling | NA | No income: 16 (72.7%) BDT 0–1000: 3 (13.6%) BDT 1001–2000: 2 (9%) BDT 2001 and above: 1 (4.5%) Household-size (Mean): 4.2 | To explore the relationship dimensions of older women with multimorbidity in homecare and their utilization of health services. | Axel Honneth recognition and misrecognition theory | qualitative case-study | Convenience Sampling based on a cohort study | semi-structured in-depth interviews | in-person | A critical thematic discourse analysis |
| 10 | A socially excluded space: restrictions on access to health care for older women in rural Bangladesh ^10^ | Hossen | Qualitative Health Research | 2010 | 2006 | Sherpur district, Bangladeshi | patients | NA | 17 | approximately 60~75 | 17 female (100%) | NA | 11 widow (64.7%), 6 lived with a spouse (35.3%) | 10 (58.8%) no formal education, among the 7 (41.2%) who did, none had completed high school | NA | All of the participants knew how to read the Quran, however, because this is required for marriage. All of them worked in their homes. | To explore the experiences of women from rural Bangladesh about decision-making processes with respect to their access to health care and whether they perceived that there were differences based on age and sex in the way a household responds to an illness episode. | a social-determinants-of-health perspective | Phenomenology (a feminist phenomenological approach) | Convenience sampling | Semi-structured interview | in-person | A thematic approach (inductive) |
| 11 | Coping Strategies of Older Rural Bangladeshi Women With Health Problems ^11^ | Hossen | Health Care for Women International | 2013 | 2006 | Sherpur district, Bangladeshi | patients | NA | 17 | approximately 60~75 | 17 female (100%) | NA | 11 widow (64.7%), 6 lived with a spouse (35.3%) | 10 (58.8%) no formal education, among the 7 (41.2%) who did, none had completed high school | NA | All of the participants knew how to read the Quran, however, because this is required for marriage. All of them worked in their homes. | To explore the strategies used by older women to cope with their ill health, in order to provide insight into how they might be drawn to use modern health services | the social-determinants-of-health framework & feminist framework | Phenomenology (a feminist phenomenological approach) | Convenience sampling | Semi-structured interview | in-person | A thematic approach (inductive) |
| 12 | Improving Access to Government Health Care in Rural Bangladesh: The Voice of Older Adult Women ^12^ | Hossen | Health Care for Women International | 2011 | 2006 | Sherpur district, Bangladeshi | patients | NA | 17 | approximately 60~75 | 17 female (100%) | NA | 11 widow (64.7%), 6 lived with a spouse (35.3%) | 10 (58.8%) no formal education, among the 7 (41.2%) who did, none had completed high school | NA | All of the participants knew how to read the Quran, however, because this is required for marriage. All of them worked in their homes. | To provide an in-depth understanding ofthe health-system-related barriers to utilization ofhealth services by older women living in rural Bangladesh | the social-determinants-of-health framework & feminist framework | Phenomenology (a feminist phenomenological approach) | Convenience sampling | semistructured interview | in-person | A phenomenological thematic approach |
| 13 | In search of healing between two worlds: the use of traditional and modern health services by older women in rural Bangladesh ^13^ | Hossen | Social Work in Health Care | 2012 | 2006 | Sherpur district, Bangladeshi | patients | NA | 17 | approximately 60~75 | 17 female (100%) | NA | 11 widow (64.7%), 6 lived with a spouse (35.3%) | 10 (58.8%) no formal education, among the 7 (41.2%) who did, none had completed high school | NA | All of the participants knew how to read the Quran, however, because this is required for marriage. All of them worked in their homes. | 1. Which health care providers are sought by older women living in a rural village in Bangladesh when they are ill? 2. What motivates them to choose traditional health care providers over modern health care providers? 3. What are the implications for health policy, specifically how should health services be delivered to engage these older women in the use of preventive and curative modern medicine? | the social-determinants-of-health framework & feminist framework | Phenomenology (a feminist phenomenological approach) | Convenience sampling | Semi-structured interview | in-person | A thematic approach (inductive) |
| 14 | Willingness to use and pay for options of care for community-dwelling older people in rural Vietnam ^14^ | Le | BMC Health Services Research | 2012 | 2007 | Bavi, Vietnam | both: Four FGDs: One group - six elderly people. One group - six representatives of households with older people (2 are caregivers) | NA | 6 | >60 | 3 females (50%) | NA | NA | NA | NA | NA | To understand perceived care needs of the elderly, current and expected roles of different key stakeholders, encouraging/limiting factors in providing needed care, solutions for overcoming barriers in providing the care, and expected future models of care. | NA | Convergent mixed method study design | NA | focus group discussions (FGD) | in person | Thematic content analysis |
| 15 | A Population-based Study of Care-seeking Behavior in Rural Tanzanians With Glaucoma Blindness ^15^ | Lewallen | Journal of Glaucoma | 2011 | 2007 | Kilimanjaro Region, Tanzania | patients | bilaterally blind (VA<3/60) from posterior segment causes, including glaucoma, other optic nerve disease, and various retinal conditions | 19 | mean: 77 (SD=15) | 8 females (42%) | NA | NA | NA | NA | NA | To obtain information that might be helpful in designing programs to decrease glaucoma blindness in future | NA | Narrative research | Multistage sampling based on screening | In-depth interviews | in-person | framework analysis |
| 16 | Using participatory action research to provide health promotion for disadvantaged elders in Shaanxi Province, China ^16^ | Liu | Public Health Nursing | 2006 | NA | Shaanxi, China | patients | NA | 20 | mean 65.72; range 60~72 | ~~11~~ females (55%) | NA | 13 married (65%), 7 widowers (35%) | 16 (80%) had completed 1–9 years of primary education, and no one had completed secondary or higher education | NA | 15 (75%) lived with children, 3 (15%) lived with spouses, and 2 (10%) lived alone. | (a) determine health promotion issues concerning disadvantaged elders, (b) identify social, economic, environmental, and personal factors that adversely influence disadvantaged elders’ health, and (c) enable various stakeholders working within a coordinated framework to promote health among elders | Participatory action research (PAR) model & critical social theory | participatory action research (PAR) | purposive sampling | Focus group discussion | in person | Content analysis |
| 17 | Accessibility and satisfaction of the elderly living in rural areas in relation to the health services ^17^ | Maciel | Acta Scientiarum - Health Sciences | 2020 | 2018 | Rio Grande do Sul, Brazil | paients | NA | 19 | ≥60 | NA | NA | NA | NA | NA | NA | To learn about the conditions of access to health services that seniors living in rural areas have, as well as their satisfaction. | NA | Descriptive and exploratory qualitative study | NA | semi-structured interviews | in-person | thematic analysis |
| 18 | What the elderly experience and expect from primary care services in KwaZulu-Natal, South Africa ^18^ | Naidoo | African Journal of Primary Health Care & Family Medicine | 2019 | 2018 | KwaZulu-Natal, South Africa | patients | NA | 28 | >60 | 19 females (67.9%) | Multiple ethnic backgrounds: black, Indian, mixed race and white | NA | NA | NA | NA | To explore the experiences and expectations of people aged 60 years and above regarding ageing and health services, and the factors that might improve the quality of primary care services for geriatric patients. | NA | Interpretative exploratory design | Purposive sampling | focus group | in person | thematic analysis (inductive) |
| 19 | Accessing diabetes care in rural Uganda: Economic and social resources ^19^ | Nielsen | Global Public Health | 2017 | 2011-2013 | Kasese District, Uganda | both | Type 2 diabetes (T2D) | 10 | 45~60: 2 ≥60: 8 | 4 females (40%) | Majority of the inhabitants in the district belong to the Bakonzo ethnic group | NA | NA | NA | SES status High:4 (40%) Medium: 2 (20%) Low: 4 (40%) | To explore the challenges of accessing and maintaining treatment for T2D in a rural African setting | Therapy management group | Case study | Purposive sampling | in-depth narrative interviews | in-person | content analysis |
| 20 | Reasons for poor cataract surgery uptake - a qualitative study in rural South Africa ^20^ | Rotchford | Tropical Medicine & International Health | 2002 |  | South Africa | patients | cataract | 20 | mean 78; range 56–88 | 13 females (65%) | Zulu | NA | Only 3 (15%) had ever been to school and only 1 (5%) was literate. | NA | NA | To understand the reasons for poor cataract surgery uptake in people with blindness or severe visual impairment in rural South Africa. | NA | Narrative research | Two-stage cluster sampling | Semi-structured interview | in person | thematic analysis |
| 21 | They 'don't cure old age': older Ugandans' delays to health-care access ^21^ | Schatz | Ageing & Society | 2018 | 2015 | Kalungu District, Uganda | patients | 2 groups with HIV; 2 groups with NCD; 5 groups without specific diagnosis | 9 groups (7~8 people in each group) | >60 | Men: 5 groups; Women: 4 groups | NA | NA | NA | NA | NA | Examining the factors that cause older Ugandans to delay health-care access. | Three-Delay Model | Narrative research | Purposive sampling | FGD | in person | thematic analysis |
| 22 | “How Would We Deserve Better?” Rural–Urban Dichotomy in Health Seeking for the Chronically Ill Elderly in China ^22^ | Long | Qualitative Health Research | 2016 | NA | Shandong, China | both | NA | patients: 12; caregivers: 12 | patients: mean 72, range 56–82; caregivers: Median (range)：53 (29–79) | patients: 7 female (58.3%); caregivers: 8 females (66.7%) | NA | patients: 6 widow (50%), 6 married (50%) ; caregivers: 12married (100%) | NA | NA | patients: Living arrangements  Alone: 5 With spouse only: 6 With children: 1 Monthly household income, Median (range):105 (40–3,000)(Yuan Number of children alive, Median (range)：4（2-5） | To understand the rural–urban dichotomous pattern of health-seeking behavior in China. | Bourdieu's dialectical model | narrative | Purposive sampling method | Semi-structured interviews | in-person | Abductive approach |
| 23 | Elderly Chinese and their family caregivers' perceptions of good care: A qualitative study in Shandong, China ^23^ | W. Li | Journal of Gerontological Social Work | 2012 | NA | Shandong, China | both | Heart disease: 3 Diabetes Stroke: 2 Bronchitis: 1 Hypertension: 1 Other:4 | patients: 12; caregivers: 12 | patients: mean 72, range 56–82; caregivers: Median (range)：53 (29–79) | patients: 7 female (58.3%); caregivers: 8 females (66.7%) | NA | patients: 6 widow (50%), 6 married (50%); caregivers: 12married (100%) | NA | NA | patients: Living arrangements  Alone: 5 With spouse only: 6 With children: 1 Monthly household income, Median (range):105 (40–3,000)(Yuan） Number of children alive, Median (range)：4（2-5） | Understand what older Chinese people with chronic illness and their family caregivers perceive to be good care, and to compare perspectives of those living in rural and urban areas. | NA | narrative | A purposive sampling method | Semi-structured interviews | in-person | Thematic content analysis |
| 24 | Multi-Dimensional Accessibility Barriers in Care Services for the Rural Elderly with Disabilities: A Qualitative Study in China ^24^ | Wang | International Journal of Environmental Research & Public Health | 2021 | 2019 | Shandong, China | patients | Living with disability | 13 | ≥60 | 7 female (53.8%) | NA | NA | NA | NA | 1) Multi-Disabled Family: Yes: 5 No: 6 Living alone: 2 2) Primary Caregivers Spouse: 8 Daoghter: 1 Spouse and daughter: 1 Son and daughter: 1 None: 2  (3)7 from a suburban village, 6 from a more traditional village | This research covers a multi-dimensional investigation into accessibility barriers in care services for older people with disabilities in rural China. | Theory of welfare pluralism | narrative | purposive sampling | In-depth interviews | in-person | thematic analysis method |
| 25 | Seeking medical services among rural empty-nest elderly in China: a qualitative study ^25^ | Wu | BMC Geriatrics | 2022 | 2020 | Shandong, China | patients | Healthy without illness 3; With minor illness 2; With mild chronic illness 4; With serious chronic illness 7 | 16 | 60~70: 8; 70~80: 6; 80~100: 2 | 8 females (50%) | NA | NA | NA | NA | "Empty-nest elderly": the elderly people who do not have children, or who do not live together with their children, and thus, they live alone or with their spouses | To understand the experiences of rural empty-nest elderly in seeking medical services in China. | NA | Narrative research | Purposive sampling | semi-structured interviews | in person | inductive content analysis |
| 26 | Understanding barriers to cataract surgery among older persons in rural China through focus groups ^26^ | Zhang | Ophthalmic Epidemiology | 2011 | NA | Guangdong, China | patients | cataract (best-corrected vision of < 6/18 in either eye) | 20 | mean 72.7 ± 6.1 years | 14 females (70%) | NA | NA | NA | NA | NA | To identify barriers to cataract surgery specific to older persons in rural Guangdong, China. | NA | Narrative research | Maximum variation sampling | Focus Group (FG) | in person | content analysis |
| 27 | Access to Care by Older Rural People in a Post-Reform Chinese Hospital: an Ethical Evaluation of Anthropological Findings ^27^ | Zou | Asian Bioeth Rev | 2019 | 2016 | Guangdong, China | both | gerontological and nursing care | 20 patients and their caregivers | >60 | NA | NA | NA | NA | New Rural Cooperative Medical Scheme (NRCMS) | NA | Examining older people’s access to care experiences in rural China by integrating anthropological investigation with ethical inquiry. | feminist ethical framework | ethnography | NA | participant observations, semi-structured interviews, and unstructured interviews | in person | ethical analysis |
| 28 | “Unworthy of Care and Treatment”: Cultural Devaluation and Structural Constraints to Healthcare-Seeking for Older People in Rural China ^28^ | Zou | International Journal of Environmental Research and Public Health | 2020 | 2016 | Guangdong, China | both | Multiple chronic health problems: 13/20 had cardiovascular disease and dementia, 4 suffered from advanced cancer or other terminally ill conditions. | 20 in all | >60 | NA | NA | NA | NA | NRCMS | NA | Examining the experiences of seeking healthcare for rural Chinese older people | The interplay of structural-focused & cultural-centered approaches | ethnography | Convenience Sampling | Observations, semi-structured interviews, and unstructured interviews | in person | Abductive analysis |

# **Supplemental Appendix 5: Results of Mixed Methods Appraisal Tool (Green: ≥80, Yellow: 66-79, Red: <50)**

| **Publication ID** | **First Author & Year** | **Title** | **Journal** | **MMAT Score (%)** |
| --- | --- | --- | --- | --- |
| 1 | Naana Agyeman, 2019 | "When someone becomes old then every part of the body too becomes old": Experiences of living with dementia in Kintampo, rural Ghana | Transcultural Psychiatry | 80 |
| 2 | Williams Agyemang-Duah, 2019 | "Let's talk about money": how do poor older people finance their healthcare in rural Ghana? A qualitative study | International Journal for Equity in Health | 80 |
| 3 | Williams Agyemang-Duah, 2019 | Barriers to formal healthcare utilisation among poor older people under the livelihood empowerment against poverty programme in the Atwima Nwabiagya District of Ghana | BMC Public Health | 80 |
| 4 | Williams Agyemang-Duah, 2019 | Factors influencing the use of public and private health care facilities among poor older people in rural Ghana | Journal of Public Health | 60 |
| 5 | Mohammad Hamiduzzaman, 2021 | The World Is Not Mine - Barriers to Healthcare Access for Bangladeshi Rural Elderly Women | Journal of Cross-Cultural Gerontology | 80 |
| 6 | Mohammad Hamiduzzaman, 2021 | “When I suffer from fever, I eat mangos.” Determinants of health-seeking beliefs and behaviors of rural older women in Sylhet, Bangladesh | Journal of Women & Aging | 80 |
| 7 | Mohammad Hamiduzzaman, 2022 | Exploring the System Determinants Associated with Senior Women's Access to Medical Care in Rural Bangladesh | Ageing International | 60 |
| 8 | Mohammad Hamiduzzaman, 2020 | Self-reported Seasonal Symptoms and Diseases and Primary Healthcare Utilization Among Rural Elderly Women in Sylhet District, Bangladesh | Journal of UOEH | 20 |
| 9 | Mohammad Hamiduzzaman, 2021 | Aging, care and dependency in multimorbidity: how do relationships affect older Bangladeshi women's use of homecare and health services? | Journal of Women & Aging | 80 |
| 10 | Abul Hossen, 2010 | A Socially Excluded Space: Restrictions on Access to Health Care for Older Women in Rural Bangladesh | Qualitative Health Research | 80 |
| 11 | Abul Hossen, 2013 | Coping Strategies of Older Rural Bangladeshi Women With Health Problems | Health Care for Women International | 80 |
| 12 | Abul Hossen, 2011 | Improving Access to Government Health Care in Rural Bangladesh: The Voice of Older Adult Women | Health Care for Women International | 60 |
| 13 | Abul Hossen, 2012 | In search of healing between two worlds: the use of traditional and modern health services by older women in rural Bangladesh | Social Work in Health Care | 60 |
| 14 | Le Van Hoi, 2012 | Willingness to use and pay for options of care for community-dwelling older people in rural Vietnam | BMC Health Services Research | 47 |
| 15 | Susan Lewallen, 2011 | A Population-based Study of Care-seeking Behavior in Rural Tanzanians With Glaucoma Blindness | Journal of Glaucoma | 60 |
| 16 | Ming Liu, 2006 | Using participatory action research to provide health promotion for disadvantaged elders in Shaanxi Province, China | Public Health Nursing | 60 |
| 17 | Pamela Kaezynski Maciel, 2020 | Accessibility and satisfaction of the elderly living in rural areas in relation to the health services. | Acta Scientiarum. Health Sciences | 40 |
| 18 | Keshena Naidoo, 2019 | What the elderly experience and expect from primary care services in KwaZulu-Natal, South Africa | African Journal of Primary Health Care & Family Medicine | 60 |
| 19 | Jannie Nielsen, 2017 | Accessing diabetes care in rural Uganda: Economic and social resources | Global Public Health | 100 |
| 20 | A. P. Rotchford, 2002 | Reasons for poor cataract surgery uptake - a qualitative study in rural South Africa | Tropical Medicine & International Health | 60 |
| 21 | Enid Schatz, 2017 | They 'don't cure old age': older Ugandans' delays to health-care access | Ageing & Society | 100 |
| 22 | Yan Long, 2016 | “How Would We Deserve Better?” Rural–Urban Dichotomy in Health Seeking for the Chronically Ill Elderly in China | Qualitative Health Research | 100 |
| 23 | Lydia W. Li, 2012 | Elderly Chinese and their family caregivers' perceptions of good care: A qualitative study in Shandong, China | Journal of Gerontological Social Work | 80 |
| 24 | Yuan Wang, 2021 | Multi-Dimensional Accessibility Barriers in Care Services for the Rural Elderly with Disabilities: A Qualitative Study in China | International Journal of Environmental Research and Public Health | 100 |
| 25 | Yijin Wu, 2022 | Seeking medical services among rural empty-nest elderly in China: a qualitative study | BMC Geriatrics | 80 |
| 26 | Mingzhi Zhang, 2011 | Understanding barriers to cataract surgery among older persons in rural China through focus groups | Ophthalmic Epidemiology | 80 |
| 27 | Xiang Zou, 2019 | Access to Care by Older Rural People in a Post-Reform Chinese Hospital: an Ethical Evaluation of Anthropological Findings | Asian Bioethics Review | 60 |
| 28 | Xiang Zou, 2020 | “Unworthy of Care and Treatment”: Cultural Devaluation and Structural Constraints to Healthcare-Seeking for Older People in Rural China | International Journal of Environmental Research and Public Health | 100 |

# **Supplemental Appendix 6: The social ecological model of health and Helmke and Levitsky’s (2004) institutional adaptation model**

The social ecological model was first developed by Urie Bronfenbrenner in the 1970s to understand human development. This model was soon adopted and widely used in the behavioral sciences and public health. This model considers the complex interplay between individual, relationship, community, organization, and broader societal factors in health-seeking behaviors, rather than just emphasizing individual characteristics, skills, and proximal social influences. Since our study aims to understand the complex interactions between patients, local health systems, and the broader socioeconomic status in rural LMICs, the social ecological model therefore aligns with our research objectives. Specifically, our coding frameworks was also inspired by the five levels of the social ecological model (Individual, interpersonal, organizational, community, as well as policy and other macrosystems).

Helmke and Levitsky's (2004) institutional adaptation model is useful for understanding people's actions in formal and informal institutions. In this article, we accept Samuel Huntington’s definition of institutions: "institutions are stable, valued, recurring patterns of behavior." ^29^. Helmke and Levitsky defined informal institutions as “socially shared rules, usually unwritten, that are created, communicated, and enforced outside of officially sanctioned channels”. By contrast, formal institutions are “rules and procedures that are created, communicated, and enforced through channels widely accepted as official, including state institutions (courts, legislatures, bureaucracies), state-enforced rules (constitutions, laws, regulations), and organization rules,” or the official rules (corporations, political parties, and interest groups) ^30-32^.

In Helmke and Levitsky’s model, the relationship between formal and informal institutions varies according to their intended outcomes and effectiveness (see table S1 below): First, formal and informal institutions are complementary when they have convergent motivations and the formal institutions are effective. Second, formal and informal institutions are accommodating when they have divergent motivations and the formal institutions are effective (i.e., Informal institutions can meet the unmet needs of formal institutions, thereby increasing the stability of formal institutions). Third, informal institutions can replace formal ones to seek the same outcomes when they have similar motivations but the formal institutions are ineffective. Last, informal institutions may ignore or violate formal institutions when their objectives compete to each other and formal institutions are weak.

The final framework of our review combined these two frameworks together (Figure S1). By this revised framework, we are trying to show that (1) The importance of the formal and informal systems is different at different levels of social ecological model. For example, while the formal institutions are dominant in the urban areas or macrosystem level (e.g., public policy), informal institutions usually work better in the rural areas or at interpersonal level. (2) the system of institutions is a multi-layer system. Different types of institutions played a dominated role in different layers, which means similar stakeholders will have different practices in different layers (or different social context). In this way, like the villagers surviving in the cracks of the formal system in our review, the real interactions between formal and informal institutions are far more complex than Helmke and Levitsky's model.

Table S1: the summary of Helmke and Levitsky's (2004) institutional adaptation model

| Intended outcomes of institutions | Effectiveness of formal institutions | |
| --- | --- | --- |
|  | Effective | Ineffective |
| Convergent | Complementary: Formal and informal institutions complement each other | Substitutive: Informal institutions can replace formal ones |
| Divergent | Accommodating: informal institutions can increase the stability of formal ones | Competing: Informal institutions can violate formal ones |

Figure S1: The original social ecological model of health (By Golden et al, 2015) ^33^


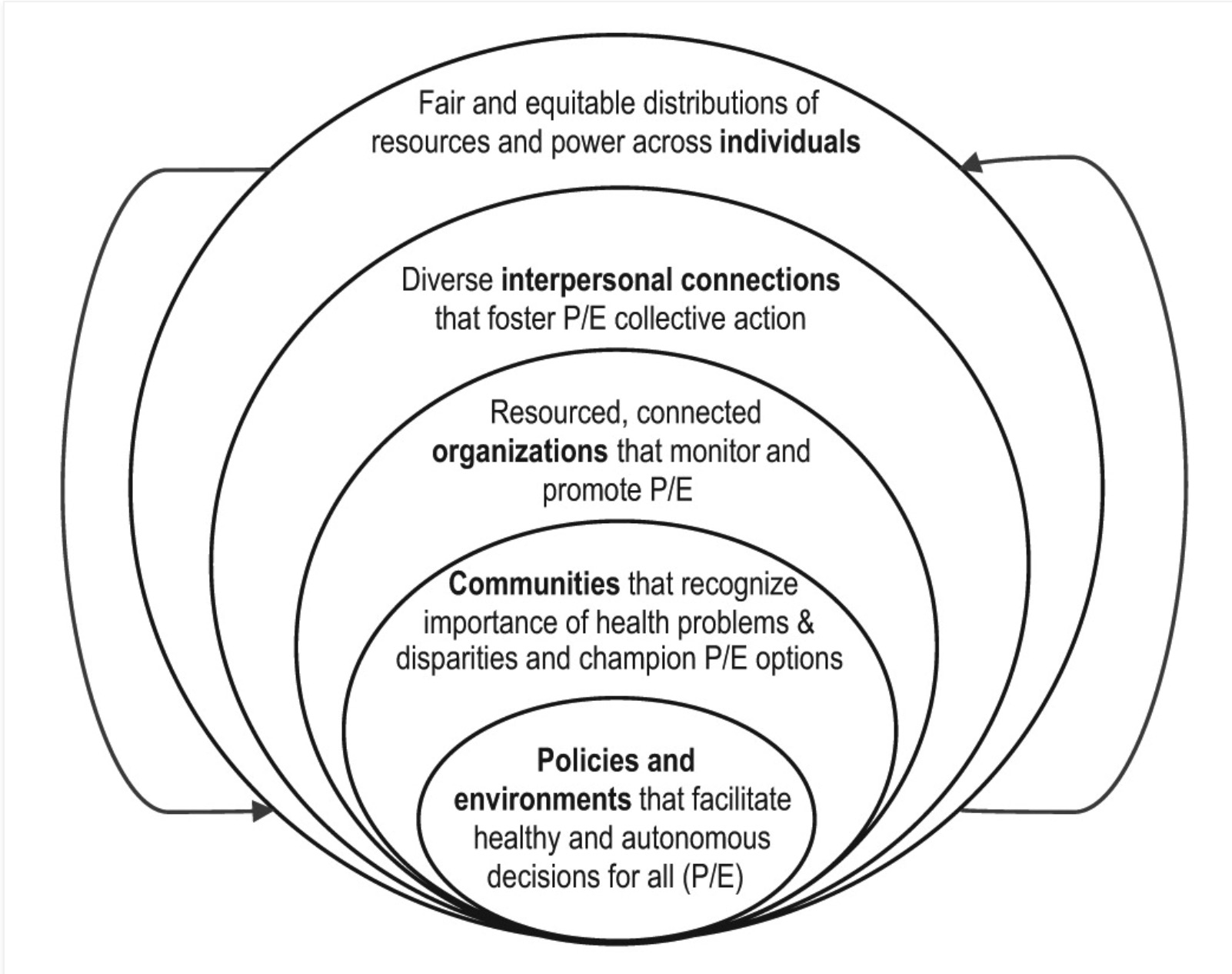


Figure S2: the analytic framework inspired by institutional adaptation model and the social ecological model of health


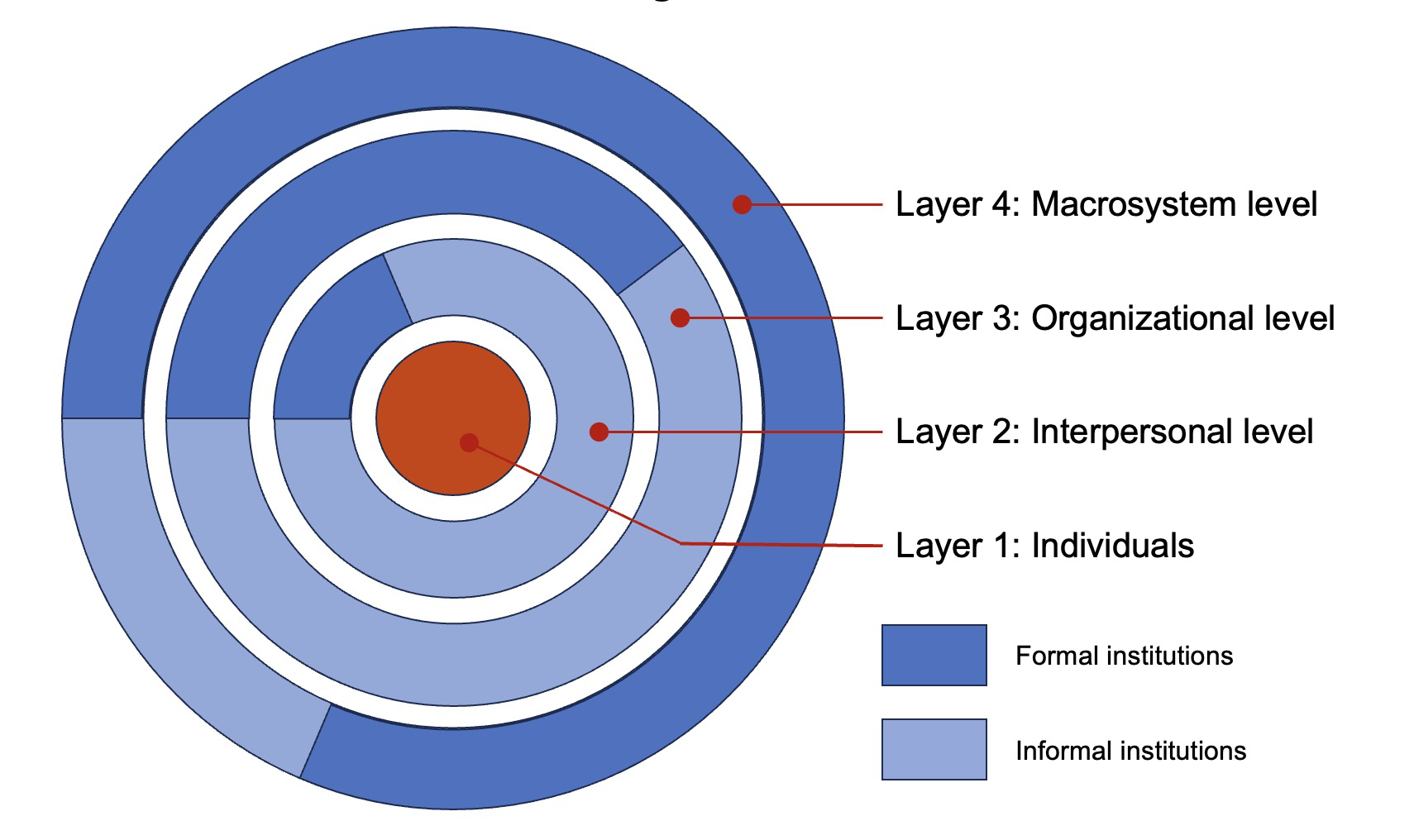


# **References**

1. Guerchet M, Nyame S, Tawiah C, et al. "When someone becomes old then every part of the body too becomes old": Experiences of living with dementia in Kintampo, rural Ghana. *Transcultural Psychiatry* 2019; **56**(5): 895-917.

2. Williams Agyemang-Duah, Charles Peprah, Peprah P. “Let’s talk about money”: how do poor older people finance their healthcare in rural Ghana? A qualitative study. *International Journal for Equity in Health* 2019; **18**(1): 1-12.

3. Agyemang-Duah W, Peprah C, Peprah P. Barriers to formal healthcare utilisation among poor older people under the livelihood empowerment against poverty programme in the Atwima Nwabiagya District of Ghana. *BMC Public Health* 2019; **19**(1): N.PAG-N.PAG.

4. Agyemang-Duah W, Peprah C, Peprah P. Factors influencing the use of public and private health care facilities among poor older people in rural Ghana. *Journal of Public Health (09431853)* 2020; **28**(1): 53-63.

5. Hamiduzzaman M, De Bellis A, Abigail W, Kalaitzidis E, Harrington A. The World Is Not Mine - Barriers to Healthcare Access for Bangladeshi Rural Elderly Women. *Journal of cross-cultural gerontology* 2021; **36(1)**: 69-89.

6. Hamiduzzaman M, De Bellis A, Abigail W, Harrington A, Fletcher A. "When I suffer from fever, I eat mangos." Determinants of health-seeking beliefs and behaviors of rural older women in Sylhet, Bangladesh. *Journal of women & aging* 2021: 1-18.

7. Hamiduzzaman M, Bellis AD, Abigail W. Exploring the System Determinants Associated with Senior Women’s Access to Medical Care in Rural Bangladesh. *Ageing International* 2022; **47**(3): 578-95.

8. Hamiduzzaman M. Self-reported Seasonal Symptoms and Diseases and Primary Healthcare Utilization Among Rural Elderly Women in Sylhet District, Bangladesh. *Journal of UOEH* 2020; **42(2)**: 175-85.

9. Hamiduzzaman M, Torres S, Fletcher A, Islam MR, Siddiquee NA, Greenhill J. Aging, care and dependency in multimorbidity: how do relationships affect older Bangladeshi women's use of homecare and health services? *Journal of women & aging* 2021: 1-14.

10. Hossen A, Westhues A. A socially excluded space: restrictions on access to health care for older women in rural Bangladesh. *Qualitative Health Research* 2010; **20**(9): 1192-201.

11. Hossen MA, Westhues A, Maiter S. Coping Strategies of Older Rural Bangladeshi Women With Health Problems. *Health Care for Women International* 2013; **34**(12): 1116-35.

12. Hossen A, Westhues A. Improving Access to Government Health Care in Rural Bangladesh: The Voice of Older Adult Women. *Health Care for Women International* 2011; **32**(12): 1088-110.

13. Hossen A, Westhues A. In search of healing between two worlds: the use of traditional and modern health services by older women in rural Bangladesh. *Soc Work Health Care* 2012; **51**(4): 327-44.

14. Van Hoi L, Thi Kim Tien N, Van Tien N, et al. Willingness to use and pay for options of care for community-dwelling older people in rural Vietnam. *BMC Health Services Research* 2012; **12**(1): 36-.

15. Lewallen S, Hassan HG, Al Attas AH, Courtright P. A population-based study of care-seeking behavior in rural tanzanians with glaucoma blindness. *Journal of Glaucoma* 2011; **20(6)**: 361-5.

16. Liu M, Gao R, Pusari N. Using participatory action research to provide health promotion for disadvantaged elders in Shaanxi Province, China. *Public Health Nursing* 2006; **23(4)**: 332-8.

17. Maciel PK, Peters CW, Lange C, Castro DSP, Braga JNR, Stolz PV. Accessibility and satisfaction of the elderly living in rural areas in relation to the health services. *Acta Scientiarum - Health Sciences* 2020; **42(1)**: 1-7.

18. Naidoo K, Van Wyk J. What the elderly experience and expect from primary care services in KwaZulu-Natal, South Africa. *Afr* 2019; **11**(1): e1-e6.

19. Nielsen J, Bahendeka SK, Bygbjerg IC, Meyrowitsch DW, Whyte SR. Accessing diabetes care in rural Uganda: Economic and social resources. *Glob Public Health* 2017; **12**(7): 892-908.

20. Rotchford AP, Rotchford KM, Mthethwa LP, Johnson GJ. Reasons for poor cataract surgery uptake - a qualitative study in rural South Africa. *Trop Med Int Health* 2002; **7**(3): 288-92.

21. Schatz E, Seeley J, Negin J, Mugisha J. They 'don't cure old age': older Ugandans' delays to health-care access. *Ageing & Society* 2018; **38**(11): 2197-217.

22. Long Y, Li LW. “How Would We Deserve Better?” Rural–Urban Dichotomy in Health Seeking for the Chronically Ill Elderly in China. *Qualitative Health Research* 2016; **26**(12): 1689-704.

23. Li LW, Long Y, Essex EL, Sui Y, Gao L. Elderly Chinese and their family caregivers' perceptions of good care: A qualitative study in Shandong, China. *Journal of Gerontological Social Work* 2012; **55**(7): 609-25.

24. Wang Y, Qi C. Multi-Dimensional Accessibility Barriers in Care Services for the Rural Elderly with Disabilities: A Qualitative Study in China. *Int J Environ Res Public Health* 2021; **18**(12): 12.

25. Wu Y, Zhang Q, Huang Y, Qiu S. Seeking medical services among rural empty-nest elderly in China: a qualitative study. *BMC Geriatrics* 2022; **22**(1): 1-10.

26. Zhang M, Wu X, Li L, et al. Understanding barriers to cataract surgery among older persons in rural China through focus groups. *Ophthalmic Epidemiology* 2011; **18**(4): 179-86.

27. Zou X, Nie J-B. Access to Care by Older Rural People in a Post-Reform Chinese Hospital: an Ethical Evaluation of Anthropological Findings. *Asian Bioethics Review* 2019; **11**(1): 57-68.

28. Zou X, Fitzgerald R, Nie JB. "Unworthy of care and treatment": Cultural devaluation and structural constraints to healthcare-seeking for older people in rural China. *International Journal of Environmental Research and Public Health* 2020; **17(6)**.

29. Huntington SP. Political order in changing societies: Yale university press; 2006.

30. Helmke G, Levitsky S. Informal institutions and comparative politics: A research agenda. *International handbook on informal governance* 2012.

31. Fukuyama F. Trust: The social virtues and the creation of prosperity: Simon and Schuster; 1996.

32. Bian Y. Guanxi, how China works: John Wiley & Sons; 2019.

33. Golden SD, McLeroy KR, Green LW, Earp JAL, Lieberman LD. Upending the social ecological model to guide health promotion efforts toward policy and environmental change. Sage Publications Sage CA: Los Angeles, CA; 2015. p. 8S-14S.

34. Lora-Wainwright A. ‘If you can walk and eat, you don’t go to hospital’: the quest for healthcare in rural Sichuan. China's Changing Welfare Mix: Routledge; 2011: 121-42.
